# Evidence for fungal and chemodenitrification based N_2_O flux from nitrogen impacted coastal sediments

**DOI:** 10.1038/ncomms15595

**Published:** 2017-06-05

**Authors:** Scott D. Wankel, Wiebke Ziebis, Carolyn Buchwald, Chawalit Charoenpong, Dirk de Beer, Jane Dentinger, Zhenjiang Xu, Karsten Zengler

**Affiliations:** 1 Department of Marine Chemistry and Geochemistry, Woods Hole Oceanographic Institution, Woods Hole, Massachusetts 02543, USA; 2 Department of Biology, University of Southern California, Los Angeles, California 90089, USA; 3 Max Planck Institute for Marine Microbiology, Bremen D-28359, Germany; 4 Department of Pediatrics, University of California, San Diego, California 92110, USA

## Abstract

Although increasing atmospheric nitrous oxide (N_2_O) has been linked to nitrogen loading, predicting emissions remains difficult, in part due to challenges in disentangling diverse N_2_O production pathways. As coastal ecosystems are especially impacted by elevated nitrogen, we investigated controls on N_2_O production mechanisms in intertidal sediments using novel isotopic approaches and microsensors in flow-through incubations. Here we show that during incubations with elevated nitrate, increased N_2_O fluxes are not mediated by direct bacterial activity, but instead are largely catalysed by fungal denitrification and/or abiotic reactions (e.g., chemodenitrification). Results of these incubations shed new light on nitrogen cycling complexity and possible factors underlying variability of N_2_O fluxes, driven in part by fungal respiration and/or iron redox cycling. As both processes exhibit N_2_O yields typically far greater than direct bacterial production, these results emphasize their possibly substantial, yet widely overlooked, role in N_2_O fluxes, especially in redox-dynamic sediments of coastal ecosystems.

## Abstract (web summary)

Predicting nitrous oxide emissions (N_2_O) remains difficult due to the numerous N_2_O production pathways. Here, the authors use incubations simulating high nitrate inputs to show that, in intertidal sediments, increases in N_2_O flux are largely mediated by fungal denitrification and/or chemodenitrification.

Nitrogen (N) loading from anthropogenic activities profoundly impacts ecosystems worldwide, with loading to coastal zones among the largest challenges facing humanity, as nearly half the global population lives within 100 km of the coast. Coastal sediments are known hotspots of biogeochemical transformations and recognized as effective agents for removing excess nitrogen^1^,^2^. However, biological removal of reactive nitrogen may also occur at the expense of increased production of nitrous oxide (N_2_O), a potent climatically active trace gas. Despite being the largest ozone depleting substance currently emitted to the atmosphere^3^, N_2_O remains unregulated by the international community and large uncertainties exist concerning N_2_O budgets (>100%), particularly for heterogeneous environments such as coasts^4^,^5^. Redox-dynamic estuarine and coastal sediments routinely experience high N loading and low dissolved oxygen (O_2_), conditions that are strongly linked to elevated N_2_O and underlie their estimated 10% contribution to the global N_2_O flux^4^,^6^,^7^,^8^,^9^. Thus, understanding their role in both nitrogen removal and N_2_O production is important for improving predictions of long-term impacts of human activity across globally relevant scales.

Many studies have focused on the relative contribution of bacterial denitrification (bDNF) or nitrification (oxidation of ammonia (NH_3_) to nitrite (NO_2_^−^) or ‘AMO'), as controlling processes underlying N_2_O emissions (Fig. 1). While yields of N_2_O from both AMO and bDNF are low (<1% in terms of total moles of N converted), their magnitude and ubiquity across ecosystems translates into major atmospheric fluxes. Increasingly, however, the potential for other N_2_O production processes has become apparent, including production of N_2_O by fungi and/or abiotic reactions coupled to redox cycling of metals such as iron^10^,^11^,^12^. In particular, the organic-rich and redox-dynamic regimes of estuarine and coastal sediments may promote both fungal activity and rapid redox cycling of iron. To examine controls and mechanisms of N_2_O production in coastal sediments (Fig. 1), we incubated natural sediment cores under flow-through conditions, manipulating both dissolved O_2_ and nitrate in the overlying water (using conditions typifying anthropogenically impacted ecosystems), while monitoring both porewater N_2_O profiles and stable isotopic fluxes of ammonium, nitrate, nitrite and N_2_O. Given the complexity of processes involved, we also leveraged the use of a less-traditional isotope system (^17^O, described below) to provide even broader perspective for disentangling operative N_2_O cycling mechanisms.

The steady-state emission flux of N_2_O (*F*_N2O_) is governed by six possible production fluxes (F) (Fig. 1; bacterial denitrification (bDNF), fungal denitrification (fDNF), chemodenitrification (cDNF; specifically the abiotic reduction of NO_2_^−^ to N_2_O by Fe(II)), ammonia oxidation by bacteria (bAMO) or archaea (aAMO) and nitrifier-denitrification (nDNF)), as well as respiratory consumption by denitrifying bacteria (N_2_O_RED_) such that:


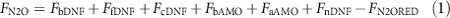


Stable isotopes of N_2_O have been widely used for studying its production and consumption, including both oxygen (^18^O/^16^O) and bulk nitrogen (^15^N/^14^N) (δ=(((*R*_sample_/*R*_standard_)−1) × 1,000) and *R*=^15^N/^14^N or ^18^O/^16^O)^13^,^14^,^15^,^16^,^17^,^18^,^19^. In addition, the unique intra-molecular distribution of ^15^N within N_2_O molecules has emerged as a powerful tool for constraining N_2_O cycling, as differences in ^15^N content between the central ‘α' and outer ‘β' atoms of the N_2_O molecule (‘site preference' or SP_N2O_, where SP_N2O_=δ^15^N^α^−δ^15^N^β^) have been shown to reflect formation pathways^13^,^14^,^16^,^18^. Numerous studies have measured the steady-state δ^15^N offset between precursor molecules (NO_3_^−^, NO_2_^−^ and NH_4_^+^) and N_2_O (‘Δδ^15^N'=δ^15^N_source_−δ^15^N_N2Obulk_), as well as SP_N2O_ values towards characterizing signature compositions for the processes in equation 1. Some compositional overlap notwithstanding, the isotopic separation of many of these endmembers has been used to distinguish their relative contribution to N_2_O production, especially nitrification and denitrification^14^,^19^,^20^. Notably, the respiratory reduction of N_2_O by denitrifying bacteria can increase the δ^15^N_bulk_ of the remaining N_2_O (decreasing Δδ^15^N values), as well as SP_N2O_ values (with a distinctive relationship between isotope effects imparted on the δ^15^N of the bulk N_2_O and its site preference, ^15^ɛ_bulk_ and ^SP^ɛ, respectively^21^,^22^) modifying mixing relationships. Nevertheless, measured values of Δδ^15^N and SP_N2O_ are quantitative integrators of the proportion of each process (which may then be modified by N_2_O reduction; see Methods), serving as independently responsive tracers for constraining production mechanisms. To further interrogate N_2_O dynamics, we also used a natural atmospherically derived NO_3_^−^ having an unusual triple oxygen isotopic composition (containing excess ^17^O) that provides novel ‘isotope space' for further resolving co-occurring processes. By comparing levels of ^17^O-excess within discrete N pools (see Methods), we are able to independently quantify the proportion of N_2_O deriving from NO_3_^−^ (or NO_2_^−^) and thereby, in concert with the more conventional isotopic measurements (Δδ^15^N, SP), uniquely explain variations in N_2_O producing pathways. Below we summarize our results, combining microprofile perspectives with the use of these mass and isotopic fluxes to constrain N_2_O production mechanisms in coastal sediments of the Wadden Sea under a variety of incubation conditions (Supplementary Fig. 1). Specifically, we find that the isotopic composition of increased N_2_O fluxes resulting from elevated nitrate loading in our incubations requires substantial contribution by processes not regularly considered in coastal ecosystems, namely fungal and/or chemodenitrification. We suggest that variations in the contribution of these processes to N_2_O fluxes from coastal and other ecosystems may help to explain the notorious variability that is frequently encountered in studies of N_2_O dynamics.

## Results

### Microsensor and mass flux perspectives on N_2_O

**Figure 2:**
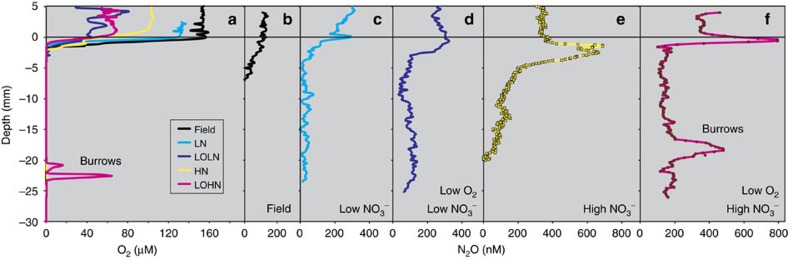
Example microsensor profiles of O_2_ and N_2_O from sediment core incubations from the Mischwatt site illustrating typical heterogeneity observed. Rapid consumption of O_2_ in the upper 2–3 mm (**a**) reflects the high organic matter content and respiration rates of these sediments. Depth profiles of N_2_O are shown in **b** through **f**. ‘Field' profile (**b**) reflects conditions immediately upon core collection. Profiles from experiment cores reflect porewater conditions after ∼7–8 days of incubation under low NO_3_^−^ (**c**), low O_2_ and low NO_3_^−^ (**d**), high NO_3_^−^ (**e**) and low O_2_ and high NO_3_^−^ (**f**). The complexity in the structure of the N_2_O profiles—including subsurface zones of production likely influenced by bioirrigation—complicate their use in conventional flux estimation by porewater models.

Microsensor profiles revealed shallow O_2_ penetration (∼2–3 mm) typical of high-respiration rates occurring in organic-rich sediments, with occasional subsurface peaks in O_2_ reflecting bioturbation/bioirrigation (Fig. 2a). N_2_O profiles revealed striking vertical and lateral heterogeneity in location, magnitude and distribution of N_2_O, reflecting the complexity of N_2_O dynamics (Fig. 2b–f). Elevated N_2_O was often observed near the sediment–water interface, suggesting oxidative production by AMO. However, zones of extremely elevated N_2_O (>3 μM, not shown) were also observed much deeper, highlighting its spatial heterogeneity. In some cases, subsurface N_2_O appeared connected with the overlying water, co-occurring near subsurface O_2_ peaks and reflecting oxidative N_2_O production despite reducing surroundings. In others, elevated N_2_O coincided with anoxic conditions, suggesting N_2_O production by reductive pathways (Fig. 2b). Several cores had active burrows extending into the sediment, likely contributing to this heterogeneity. In part, these observations extend the view of N_2_O dynamics to slightly deeper sediment layers, in contrast to previous observations mostly focusing on dynamics in the upper 1 cm (refs 23, 24). This remarkable heterogeneity, often even laterally within a single core, hinders straightforward N_2_O flux calculations, yet emphasizes the utility of the whole core incubation stable isotope approach, which integrates this natural heterogeneity and provides a complementary and mechanistic perspective on underlying N_2_O dynamics.

In contrast to the oxidative N_2_O production captured by the micro-profiles at the sediment–water interface, mass fluxes suggest an important role for reductive N_2_O production. Nitrate levels in the overlying water were closely related to N_2_O flux from the sediment, with net efflux of N_2_O from the sediment in all cases ranging from 1.4 up to 84.2 μmoles m^−2^ d^−1^ (Table 1; Supplementary Fig. 1; Supplementary Table 2). While fluxes of N_2_O under low nitrate conditions averaged 17.8 μmoles m^−2^ d^−1^ (Table 1), decreasing dissolved O_2_ saturation to ∼30% reduced N_2_O fluxes (5.9 μmoles m^−2^ d^−1^). In contrast, addition of NO_3_^−^ to the overlying water significantly increased N_2_O (as well as NO_2_^−^) fluxes (Table 1) to the water column, consistent with other studies linking elevated coastal NO_3_^−^ loading with efflux of N_2_O to the atmosphere^6^,^8^,^25^. Corresponding NO_3_^−^ consumption also increased significantly, reflecting diffusion-limitation of organic matter respiration (Table 1; Supplementary Fig. 1; Supplementary Table1).

### Multi-isotope analysis of underlying N_2_O cycling processes

**Figure 3:**
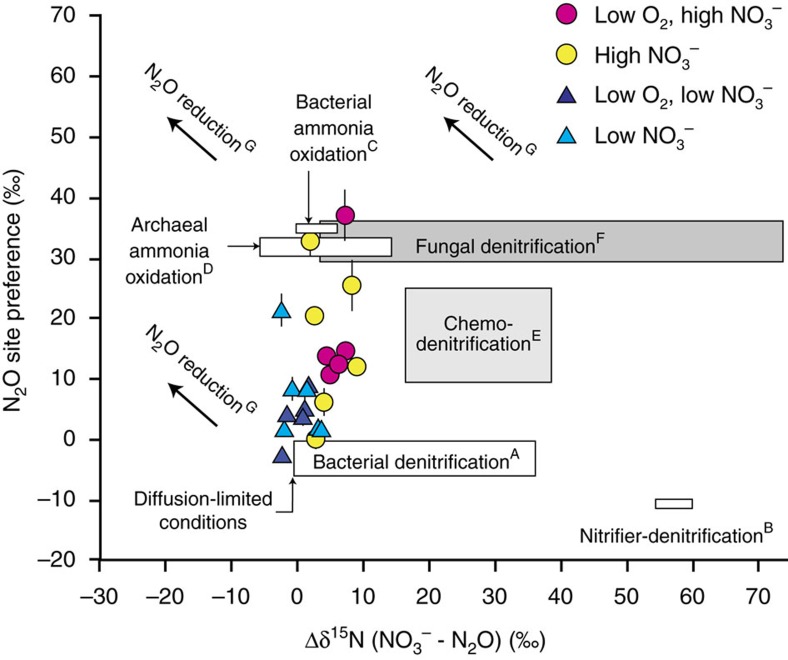
Multiple isotope plot of N_2_O illustrating predicted compositional fields of various processes as well as steady-state composition of N_2_O fluxes from incubations. For illustrating representative compositional fields of N_2_O production processes (boxes), ranges are taken from literature reports of the offset between precursor molecule and predicted N_2_O (Δδ^15^N, or the difference in δ^15^N between NH_4_^+^ and N_2_O for bacterial and archaeal ammonia oxidation, between NO_2_^−^ and N_2_O for chemodenitrification or between NO_3_^−^ and N_2_O for denitrification). References (in parentheses) include for (A) bacterial denitrification^20^,^83^,^84^, (B) nitrifier-denitrification^59^, (C) bacterial ammonia oxidation^20^,^59^,^84^,^85^, (D) archaeal ammonia oxidation^56^,^57^,^58^, (E) chemodenitrification^47^,^48^,^49^, (F) fungal denitrification^37^,^42^,^43^,^44^ and (G) bacterial N_2_O reduction^21^,^22^,^86^, which has been shown to result in a coupled increase in δ^15^N and SP of the remaining N_2_O pool (indicated by arrows). Experimental *x*-axis data are δ^15^N_NO3_–δ^15^N_N2O_. Error bars in the *y*-direction are 1 s.d., while in the *x*-direction error is smaller than the symbols. Note that for mass balance calculations the actual measured steady-state isotope values of precursor molecules (for example, NH_4_^+^ or NO_3_^−^) were used as appropriate for constraining endmember values of each process. In general, addition of ∼100 μM NO_3_^−^ to the overlying water increased N_2_O fluxes with an accompanying increase in N_2_O site preference, while not decreasing the Δδ^15^N (as would be expected from N_2_O reduction, arrows), implicating an increased production of N_2_O from a process deriving from NO_3_^−^ (or NO_2_^−^) yet also one yielding an elevated site preference such as chemodenitrification and/or fungal denitrification.

The stable isotopic composition of N_2_O and other nitrogen pools (Table 2; Supplementary Table 2) provides additional insight into specific biogeochemical mechanisms regulating sedimentary N_2_O fluxes. Average SP_N2O_ for low-nitrate (LN) and low-O_2_/low-nitrate (LOLN) experiments were not significantly different (7.2±3.4 and 6.2±3.2‰, respectively) and, together with small differences between effluent NO_3_^−^ and N_2_O δ^15^N (Δδ^15^N) indicate that N_2_O fluxes were largely linked to bacterial denitrification (having low SP_N2O_) that was limited by diffusive supply of NO_3_^−^ (yielding low Δδ^15^N) (Fig. 3), a common characteristic of organic-rich sediments^26^. Notably, SP_N2O_ values were higher than values expected from bDNF alone, however, reflecting contribution by additional N_2_O cycling processes.

In contrast, the increased N_2_O fluxes under elevated NO_3_^−^ exhibited higher SP_N2O_ relative to low nitrate experiments, averaging 16.2±5.0 and 12.9±2.5‰ for the high-nitrate (HN) and low-O_2_/high nitrate (LOHN), respectively (Fig. 3, Table 2), and indicating a shift in N_2_O dynamics. This increase in SP_N2O_, however, was not accompanied by an increase in δ^15^N of the N_2_O pool (no apparent decrease in Δδ^15^N), as would be expected by increased N_2_O reduction^21^,^27^. This observation implicates the stimulation of processes that produce N_2_O with high SP_N2O_—namely, b/aAMO, fDNF and/or cDNF. Below, we use the triple oxygen isotopes of co-existing NO_3_^−^, NO_2_^−^ and N_2_O to further examine these candidate processes responding to elevated NO_3_^−^ loading.

### Triple oxygen isotopes as a tool for constraining nitrogen cycling

Our multi-pool Δ^17^O measurements enable disentangling of processes that are otherwise overlapping (in SP_N2O_ values, for example), providing a complementary perspective to the N isotope analyses. First, these analyses revealed that nitrification played a relatively small role in NO_3_^−^ production. As noted, amended nitrate had a high Δ^17^O_NO3_ value (+18.5‰), which when combined with pre-existing nitrate in the supply seawater (Δ^17^O_NO3_=0‰) yielded a Δ^17^O_NO3_ of ∼ +15.3‰ (Table 2). As changes to this Δ^17^O_NO3_ value occur only by production of new nitrate during nitrification^28^,^29^, the small differences in Δ^17^O_NO3_ between the inflow and effluent (at most 0.8‰; Table 2), reflect small relative contribution of newly produced NO_3_^−^ by nitrification.

**Figure 4:**
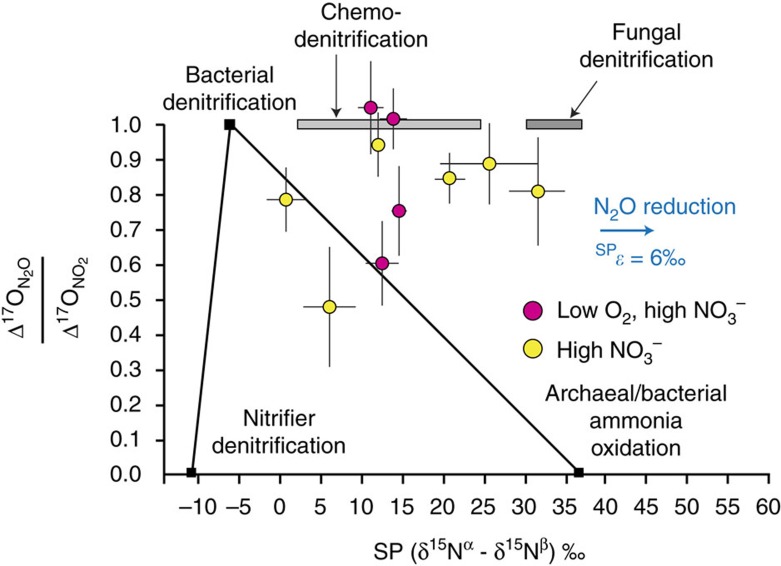
Comparison of steady state triple oxygen isotope composition of N_2_O and NO_2_^−^ with SP_N2O_ for experiments amended with NO_3_^−^. Endmember composition for the *y*-axis was based on the assumption that bacterial, fungal and chemodenitrification are utilizing the steady-state NO_2_^−^ pool as a substrate (where Δ^17^O_NO2_=steady-state value), while nitrifier-denitrification uses only NO_2_^−^ derived directly from NH_4_^+^ oxidation (Δ^17^O_NO2_=0‰). Increases in SP_N2O_ values may be caused by N_2_O reduction (Ostrom *et al*.^21^; Jinuntuya-Nortman *et al*.^86^), however, this mass dependent process will only impact SP_N2O_ and not alter Δ^17^O values (horizontal arrow). Error bars represent±1 *σ* for SP_N2O_ and propagated error for Δ^17^O_N2O_/Δ^17^O_NO2_. Data demonstrate that associated increases in SP_N2O_ upon elevated NO_3_^−^ is not associated with ammonia oxidation, but instead is linked to increased contribution of fungal and/or chemodenitrification.

Comparatively, Δ^17^O_N2O_ values averaged +6.5 and +5.4‰ for high nitrate (HN) and low O_2_/high nitrate (LOHN), respectively. These are lower than the corresponding Δ^17^O_NO3_ (Table 2), yet unequivocally reflect transfer of NO_3_^−^ derived O atoms into the N_2_O flux. Whether the lower Δ^17^O_N2O_ (relative to Δ^17^O_NO3_) stems from production of N_2_O by processes having a precursor other than NO_3_^−^ (for example, bAMO/aAMO) or from the equilibration of intermediate NO_2_^−^ with water (also causing Δ^17^O to approach 0‰) cannot be ascertained by comparing Δ^17^O_NO3_ and Δ^17^O_N2O_ (equation 2). However, Δ^17^O_NO2_ values were also typically lower than steady-state Δ^17^O_NO3_, averaging +8.5 and +9.2‰, respectively (Table 2). While non-zero Δ^17^O_NO2_ values reflect reduction of NO_3_^−^ to NO_2_^−^ (since Δ^17^O is conserved), Δ^17^O_NO2_ values lower than steady-state Δ^17^O_NO3_ values must reflect NO_2_^−^ production by ammonia oxidation (bAMO or aAMO) and/or partial equilibration of NO_2_^−^ with water. Regardless, comparison of steady-state Δ^17^O_N2O_ with Δ^17^O_NO2_ (equation 5) indicates an average of 70–80% of the N_2_O derived from a NO_2_^−^ precursor under high NO_3_^−^ incubations and thus indicates that the increased production occurred via reductive pathways (Fig. 4) and not by a/bAMO. Together with the elevated SP_N2O_ and only small changes in Δδ^15^N, this suggests fungal and/or chemodenitrification as possible contributors (Fig. 4). Both fDNF and cDNF are dependent on supply of NO_2_^−^ and typically exhibit yields far greater than bacterial N_2_O production (that is, the relative amount of N_2_O emitted per mole of NO_3_^−^ or NO_2_^−^ reduced or NH_3_ oxidized). Thus, only small levels of these processes would be required to contribute relatively large amounts of N_2_O—setting the stage for a potentially important role for these biogeochemical processes in regulating N_2_O fluxes wherever they occur.

## Discussion

Diversity and abundance of fungi in oxygen-depleted coastal sediments is generally thought to represent a small fraction of their soil-hosted counterparts^30^,^31^,^32^. Nevertheless, their ecological role remains unclear—with recent studies challenging the perspective that fungi are only ecologically significant under aerobic conditions^31^,^32^,^33^. Adapted for organic-rich environments often depleted in O_2_, many fungi have a range of cellular adaptations to life under suboxic conditions^32^,^34^,^35^,^36^, including the ability to couple denitrification^37^ directly to mitochondrial respiration^38^—a metabolic capacity that has been documented in a variety of environments including coastal sediments^34^,^39^. Given this respiratory flexibility, fDNF is poised to be especially important under hypoxic conditions and wetland environments, where access to O_2_ in overlying water is juxtaposed with anoxic, carbon-rich conditions^40^. The most characteristic feature of the fungal-denitrifying system is a P450 cytochrome operating as a nitric oxide reductase (P450nor)^41^ giving rise to the characteristically high SP of ∼35–37‰ (refs 42, 43), the biochemical nature of which was recently interrogated in the purified enzyme^44^. Although assessment of fungal metabolic activity was beyond our scope, sequencing of the fungal ITS region revealed the presence of fungi across all incubations and study sites (Supplementary Fig. 3), indicating diverse sequences including members of several divisions, some of which include N_2_O producing isolates^37^. While their presence does not confirm specific physiological activity involved in nitrogen transformations, taken together our results point to an unexplored role of fungi in coastal sedimentary N cycling. In particular, as fungi lack N_2_O reductase^38^, yields from fDNF are also typically 1–2 orders of magnitude greater than for bDNF (generally <0.1%) and N_2_O production appears to be physiologically widespread among fungi^37^. Indeed the importance of fungi in contributing to N_2_O production is well-recognized across a range of terrestrial ecosystems^45^. While their overall role in the reductive elimination of reactive N may be small relative to that of bacterial denitrification, these high yields mean that even small levels of fDNF could have disproportionately large impacts on N_2_O release, serving as an important, yet under-recognized source to the atmosphere.

Although biological N_2_O production has received much attention, abiotic production of N_2_O is also widely documented, typically via reactions involving intermediates such as NH_2_OH and NO_2_^−^—though its environmental role remains unclear (Zhu-Barker *et al*.^11^, and references therein). Specifically, reduced iron (Fe(II)), especially mineral or surface-bound Fe(II), is an effective catalyst of NO_2_^−^ reduction under a range of conditions^46^, and the presence of mineral surfaces and elevated levels of Fe(II) has also been shown to increase N_2_O yield^47^,^48^. Although data are limited, SP_N2O_ from chemodenitrification is generally >10‰ and recent evidence suggests that elevated reaction rates, promoted by high levels of Fe(II), may also increase SP_N2O_ (up to 26‰) (47,48,49,50,51). The production of reactive Fe(II) as the result of direct or indirect microbial activity is a ubiquitous feature of marine sediments. Our sites contained between 67 and 1344 μM HCl-extractable Fe(II) g^−1^ wet sediment (Supplementary Fig. 4). However, prediction of reaction kinetics between Fe(II) and NO_2_^−^ in these porewater environments is complex, particularly given the range of binding environments of Fe(II), which largely controls its reactivity^52^,^53^. Nonetheless, the positive flux of NO_2_^−^ together with the porewater Fe(II) levels suggests that cDNF may also have contributed to N_2_O production. Interestingly, however, despite its lower Fe(II), the sandy site (SD) exhibited similar overall N_2_O isotope dynamics to the other two more Fe-rich sites (Table 2), suggesting that perhaps the increased response of N_2_O production to NO_3_^−^ loading was perhaps not as tightly linked to Fe(II) content.

On the basis of the isotope systematics described, we use an isotope mass balance (based on equations 1, 2, 5 and 6 and defined endmember compositions (Supplementary Table 3)) to estimate relative contribution of operative N_2_O producing mechanisms (see Methods). While fDNF and cDNF are not mutually exclusive, we consider them separately to more robustly evaluate their potential contribution. Previous studies appear to demonstrate a strong relative dominance of ammonia oxidizing bacterial abundance compared to archaea in organic-rich coastal sediments^54^,^55^. An assumed numerical dominance of bacterial ammonia oxidizers notwithstanding, pure culture studies of archaeal ammonia oxidizers typically produce N_2_O reflecting a isotopic compositional mixture of both the AMO and nDNF pathways^56^,^57^,^58^, as has been more directly characterized in bacterial ammonia oxidizers^59^. Ongoing studies of N_2_O production mechanisms in ammonia oxidizing archaea will undoubtedly provide more insight on their unique biochemical nature. Differences in biochemistry aside, however, given the apparent similarity in isotopic composition of N_2_O deriving from bAMO and aAMO (especially a high SP_N2O_ value, Figs 3 and 4), here we opt to combine bacterial and archaeal AMO for consideration in our mass balance analysis—setting endmember values to those previously determined for bAMO, as these have been studied in far more detail^59^. Thus, for elevated nitrate experiments (in which we can leverage the use of the positive Δ^17^O), we consider N_2_O production by bDNF, nDNF and AMO (bAMO+aAMO) together with either fDNF or cDNF (Supplementary Table 4), while also examining the relative influence of N_2_O reduction on the calculated steady-state contributions of each process (see Methods). Error was estimated using a Monte Carlo approach in R with 10,000 simulations (Supplementary Table 4; see Supplementary Information for a more detailed sensitivity analysis of endmember composition).

**Figure 5:**
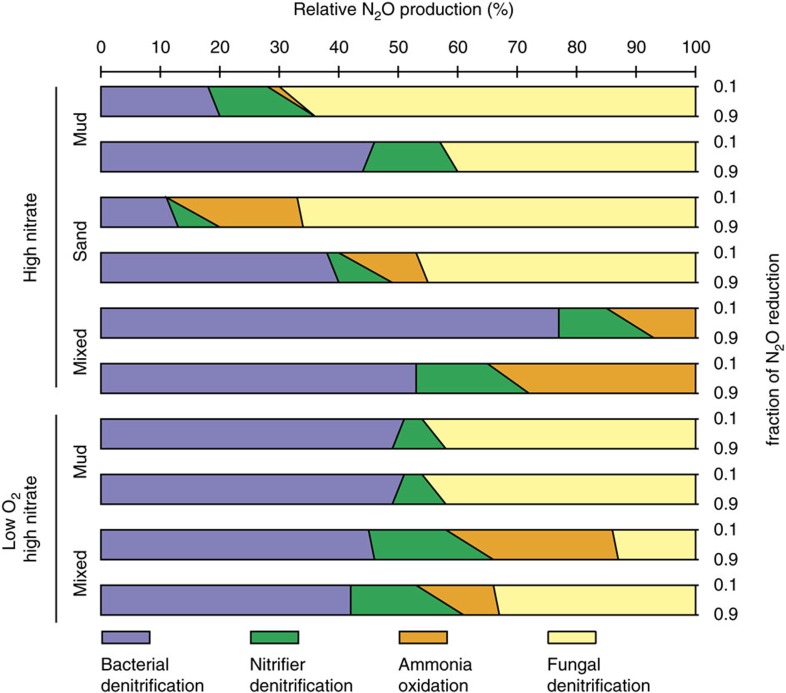
Relative contribution of N_2_O produced by four cycling processes as calculated by N_2_O isotope mass balance. Each bar reflects steady-state calculations using measurements from a single core incubation (two cores were incubated per site per treatment and are shown separately), considering bacterial denitrification, ammonia oxidation, nitrifier denitrification and fungal denitrification. For illustration of the relative influence that N_2_O reduction (and its associated isotope fractionation) plays in the mass balance estimates, scenarios of both low (10%, upper) and high (90%, lower) relative N_2_O consumption are considered for each core (right side axis). Contribution by processes where mass balance estimates yielded small negative values (Supplementary Table 4) were considered equal to zero for this figure, with other values weighted accordingly to sum to 100%. The reader is also referred to Supplementary Table 4 for associated error in mass balance estimates calculated by Monte Carlo simulation, as well as Supplementary Tables 5 and 6 for sensitivity analyses of alternative endmember compositions.

Under elevated nitrate, mass balance indicates N_2_O production predominantly driven by varying contribution of bacterial and fungal denitrification (Fig. 5; Supplementary Table 4). For the base case (Supplementary Table 3), fungal denitrification contributed on average 36% of the N_2_O flux (up to 70% in one core). In evaluating the sensitivity of these estimates to endmember composition, this average value increased to 41 or 56% (if using a lower SP value of 30.3‰ for fDNF or a lower Δδ^15^N value for the nDNF endmember, respectively; Supplementary Table 5) or decreased to 28% (if all nDNF derived N_2_O were to originate from NO_2_^−^ having a positive Δ^17^O value instead of 0‰; Supplementary Table 6). In contrast, ammonia-oxidation contributed on average only 3–12% (via NH_2_OH decomposition) and 8–17% (via nDNF) for the base case. Consideration of cDNF (in lieu of fDNF) as the endmember having both a high SP_N2O_ and a NO_2_^−^ precursor required an even higher proportion of this process to satisfy mass balance (Supplementary Table 4). However, two cores in this case exhibited isotopic compositions violating mass balance (those with highest SP_N2O_), evidently requiring at least some contribution of fDNF (having a higher endmember SP_N2O_) over cDNF. Although the LN and LOLN treatments did not involve the Δ^17^O approach, the statistically higher SP_N2O_ values under elevated nitrate (relative to low nitrate; Fig. 3, Supplementary Table 2) point to a shift in N_2_O production mechanisms in response to NO_3_^−^, which must have included increased contribution by fDNF and/or cDNF. Ultimately, while the precise contribution of N_2_O pathways varies depending on prescribed endmember compositions, all scenarios indicated substantial contribution by these non-traditional N_2_O production pathways.

Increased N_2_O emissions from coastal systems receiving elevated NO_3_^−^ are well documented^4^,^8^,^9^ and the ‘central role' of NO_2_^−^ in relation to N_2_O has been emphasized by others^6^. For example, large increases in N_2_O from sediments amended with NO_2_^−^ (relative to NO_3_^−^) was previously interpreted as evidence for ‘obligate nitrite-denitrifying bacteria' that reduce NO_2_^−^ to N_2_O (ref. 6). Similarly, based on SP_N2O_ it was concluded that N_2_O production in estuarine sediments was controlled by an as yet ‘unidentified process'^60^ having an isotopic composition consistent with more recent studies of fungal and chemodenitrification. On the basis of our results, we suggest that these previously ‘missing' and/or ‘unidentified' pathways likely represent non-traditional pathways including denitrification catalysed either by fungi or reactions involving Fe(II).

To the degree that our sediment incubations reflect processes ongoing under natural conditions, elevated NO_3_^−^ loading to coastal sediments appears to increase N_2_O fluxes largely through reactions involving a NO_2_^−^ intermediate, yet also exhibiting elevated SP values. This combination of characteristics pinpoints an increased involvement of processes not regularly considered in coastal ecosystems—namely fungal and chemodenitrification. We suggest that both may represent important, yet under-appreciated sources regulating N_2_O fluxes from redox-dynamic, organic-rich environments and warrant further examination. Studies are frequently challenged by the dynamic nature of N_2_O fluxes, which are often episodic and difficult to link to specific factors or processes (for example, refs 23, 25). Although our study was conducted at steady-state (enabling our assessment of fDNF and cDNF), we posit that the commonly observed patchy and dynamic nature of N_2_O fluxes may stem from a complex network of differential contribution by direct and indirect, biological and abiotic processes, including the metabolic activity of fungi and biogeochemical redox cycling of iron. In particular, compared to bacterial denitrification and/or ammonia oxidation, their especially high yields poise these processes to be important, yet under-recognized, contributors to N_2_O dynamics in many systems.

## Methods

### Study site and experimental setup

Twenty-four sediment cores were collected in August of 2013, from three intertidal sites near Königshafen on the island of Sylt in the North Sea, Germany. Sites were ∼100 m apart and chosen based on qualitative differences in sediment grain size and location characteristics. The ‘Schlickwatt (MD)' and ‘Mischwatt (MX)' sites were located inside a small lagoon, while the ‘Sandwatt (SD)' site was more openly exposed to wind and waves (Supplementary Fig. 1). Thirty intact push cores (30 cm length, 10 cm OD, 1/8″ wall thickness) were taken using polycarbonate core liners having vertical lines of silicone sealed holes (ø 3 mm) at 1-cm intervals to allow porewater collection using Rhizon samplers. Cores were retrieved leaving ∼10 cm of overlying water and sealed with double o-ring Delrin caps to minimize gas exchange during transport, and brought immediately back to the laboratory. In addition to the cores used for the incubations, two additional cores were used from each site for immediate microsensor profiling (O_2_, N_2_O) and pore-water extraction (‘field cores'). The remaining cores were prepared in parallel for incubations. On completion of the incubations, microsensor profiling of O_2_ and N_2_O was conducted immediately followed by extraction of porewaters.

### Incubations

The gas-tight sealed sediment cores were incubated in the dark at *in situ* temperatures (19 °C) in a temperature-controlled room at the Alfred Wegener Institute—Waddensee Field Station. Throughout the incubations the overlying water of the cores was continuously supplied with filtered seawater from large carboys, which were refilled as needed. The o-ring sealed core tops contained inlet/outlet fittings for continual delievery of fresh seawater through gas impermeable PEEK tubing (1/8″ OD). Peristaltic pumps were used to regulate flow rates at 1.8±0.06 ml min^−1^ (measured gravimetrically at each sampling point) for ∼8 days. The inflow line was placed near the sediment–water interface to minimized stratification. For experimental manipulations, four different inflow seawater compositions were used: ‘Low nitrate' (air sparged; ∼20 μM; LN), ‘Low oxygen, low nitrate' (sparged with N_2_ to 30–35% O_2_ saturation; ∼20 μM; LOLN), ‘High nitrate' (amended with NaNO_3_ to ∼120 μM (above background nitrate); HN) and ‘low oxygen, high nitrate' (combined treatments; LOHN).

### Sample collection

Samples of each sediment core effluent were taken twice per day. For dissolved ions, effluent was directed into HDPE bottles and allowed to fill for ∼60 min before subsampling, filtering (0.2 μm) and freezing (−20 °C). Separate 20 ml aliquots were taken for measurement of dissolved inorganic nitrogen concentrations (nitrate, nitrite and ammonium) and stable isotopic composition. Concentrations of nitrite and ammonium were made immediately (see below), while nitrate concentrations were measured later in the Wankel lab at WHOI. Samples for dissolved N_2_O were directed through gas impermeable PEEK tubing directly into pre-evacuated Tedlar gas sampling bags followed by gentle transfer into 160 ml serum bottles using a ¼″ OD silicone tubing, filling from the bottom to minimize turbulence and gas exchange. Sample water was allowed to overflow the bottle volume for at least two volumes before crimp-sealing with grey butyl septa and preserving with 100 μl of a saturated HgCl_2_ solution.

### Porewater sampling

Pore water samples were collected from sediment cores in 1-cm depth intervals using Rhizons^61^, which were inserted into intact sediment cores through silicon-filled ports in the walls of the core tubes. Samples of 5–10 ml volume were taken starting at the sediment–water interface down to 16 cm depth and frozen immediately for later analysis. Parallel cores were sectioned in 1-cm intervals for the analyses of iron. HCl extractable Fe(II) and the amorphous, poorly crystalline fraction of the Fe(III) minerals were measured by procedures described in ref. 63, with the modifications as in ref. 64.

### Concentration and flux measurements of N bearing species

Concentrations of NO_3_^−^+NO_2_^−^ were measured by chemiluminescence after reduction in a hot acidic vanadyl sulfate solution on a NOx analyser^65^. Concentrations of NO_2_^−^ were quantified by using the Griess–Ilosvay method followed by measuring absorption 540 nm, and NO_3_^−^ was quantified by difference^66^. Concentrations of NH_4_^+^ were measured by fluorescence using the OPA method^62^. Concentrations of N_2_O were made using the integrated peak area of the m/z 44 beam on the IRMS (see below), standardizing to analyses of known amounts of N_2_O (injected into N_2_ sparged seawater in 160 ml serum bottles) and normalizing to sample volume (158 ml). Mass fluxes (Supplementary Fig. 2; Supplementary Table 1) were calculated as a function of the steady-state difference (usually after ∼3 days) between influent and effluent concentrations (Δ[*C*]), flow rate (*r*) and sediment surface area (*A*) using: Flux=(Δ[*C*] × *r*)/*A*. Error estimates of fluxes incorporate variations in both measured flow rates as well as steady state concentrations.

### Isotope measurements

All N and O isotopic composition measurements (δ^15^N and δ^18^O; where δ^15^N=((^15^R_sample_/^15^R_Air_)−1) × 1,000 in units of ‰, and ^15^R=^15^N/^14^N and where δ^18^O=((^18^R_sample_/^18^R_VSMOW_)−1) × 1,000 in units of ‰, and ^18^R=^18^O/^16^O) were made after conversion of analytes to nitrous oxide, followed by purification with a customized purge and trap system and analysis on a continuous flow IsoPrime 100 isotope ratio mass spectrometer (IRMS).

*Nitrate*. Nitrate was converted to N_2_O using the denitrifier method^67^,^68^ after removal of nitrite by addition of sulfamic acid^69^. Corrections for drift, size and fractionation of O isotopes during bacterial conversion were carried out using NO_3_^−^ reference materials USGS 32, USGS 34 and USGS 35 (refs 67, 70), with a typical reproducibility of 0.2 and 0.4‰ for δ^15^N and δ^18^O, respectively, in the course of single run. Triple oxygen isotope measurements allow for the determination of ‘anomalous ^17^O,' (the deviation from the terrestrial fractionation line) with the magnitude of this anomaly expressed as Δ^17^O (after^71^), where:


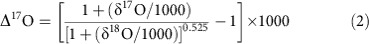


Nitrate Δ^17^O measurements were made on separate aliquots by routing denitrifier-produced N_2_O through a gold tube (1/16″ OD) held at 780 °C, thermally decomposing the N_2_O into N_2_ and O_2_, which were chromatographically separated using a 2 m column (1/16″ OD) packed with molecular sieve (5 Å) before analysis on the IRMS^72^,^73^. Nitrate reference materials USGS 35 and USGS 34 were used to normalize any scale contraction during conversion, with reproducibility of Δ^17^O typically ±0.8‰.

*Nitrite*. All samples for nitrite N and O isotope measurements were converted to N_2_O within 2 h of collection using the azide method^74^. Parallel conversions of internal nitrite standards (WILIS 10, 11 and 20) were conducted to assess potential changes in reagents with time. Internal nitrite standards were also used correct for any variations due to peak size linearity and instrumental drift, with a typical reproducibility for both δ^15^N and δ^18^O of ±0.2‰. On the basis of calibrations against isotope reference materials USGS 32, 34 and 35 for δ^15^N (ref. 75) and N23, N7373 and N10129 for δ^18^O (ref. 76), the values of WILIS 10, 11 and 20 are reported here to be −1.7, +57.1 and −7.8‰ for δ^15^N and +13.2, +8.6 and +47.6‰ for δ^18^O, respectively. Nitrite Δ^17^O measurements were made after conversion to N_2_O using the azide method and normalized using a combination of NO_2_^−^ and NO_3_^−^ isotopic reference materials. Δ^17^O values of NO_2_^−^ isotope standards WILIS 10 and WILIS 11 were calibrated previously against USGS 34 and USGS 35 using the denitrifier method followed by thermal decomposition of N_2_O to N_2_ and O_2_ as described above—yielding Δ^17^O values of 0‰ for both. For sample NO_2_^−^, raw δ^17^O and δ^18^O values were first normalized for oxygen isotopic exchange with water during the azide reaction^74^ using the calibrated δ^17^O and δ^18^O values of WILIS 10 and WILIS 11. During the same IRMS run, N_2_O produced from USGS 34 and USGS 35 via the denitrifier method was also thermally converted and analysed as N_2_ and O_2_. Because any isotope fractionation occurring during these reactions is mass dependent (Δ^17^O is unaffected), the Δ^17^O of NO_2_^−^ can be calculated by normalizing to Δ^17^O values of these NO_3_^−^ standards. We disregard the small amount of oxygen isotope exchange occurring during the denitrifier method, as this would have only a small impact on the calculated Δ^17^O values.

*Reduced nitrogen*. Total reduced nitrogen (TRN=DON+NH_4_^+^) was measured in a subset of incubation cores by oxidation of the total dissolved nitrogen (TDN) pool to nitrate via persulfate digest—followed by δ^15^N analysis using the denitrifier method^77^. The δ^15^N of the TRN pool was then calculated by mass balance by subtracting the molar contribution of the measured δ^15^N of NO_3_^−^ and NO_2_^−^ pools to the TDN pool. On the basis of the measurement of NH_4_^+^ concentrations, the DON flux was generally of the same magnitude as the NH_4_^+^ flux (not shown). For use in the mass balance calculations (for estimation of the bAMO endmember Δδ^15^N value), the δ^15^N of the TRN pool was assumed to be a reasonable proxy for the δ^15^N of the NH_4_^+^ pool. In general, this assumption had only a very small impact on the apportionment N_2_O sources by mass balance (<1%).

*Nitrous oxide*. For dissolved N_2_O, samples were extracted from the 160 ml serum bottles using a purge and trap approach^78^. Liquid samples were quantitatively transferred from the sample bottle into a purging flask using a 20 psi He stream, followed by He-sparging (∼45 min) and cryogenic trapping using the same system described above for nitrate and nitrite derived N_2_O. Isotopic composition of the dissolved N_2_O was measured by direct comparison against the N_2_O reference tank, as no isotopic reference materials were available at the time of the analyses (USGS 51 and USGS 52 have since been publicly released: http://isotopes.usgs.gov/lab/referencematerials.html). The composition of this tank (δ^15^N^bulk^=−0.7‰; δ^18^O=+39.1‰; SP_N2O_=−5.3‰, where SP_N2O_=δ^15^N(α)−δ^15^N(β) and α and β refer to the central and outer N atoms in the linear N_2_O molecule, respectively) was calibrated directly against aliquots of two previously calibrated N_2_O tanks from the Ostrom Lab at Michigan State University, having been calibrated by Tokyo Tech (Ostrom, pers. comm.). Several sample analyses of tropospheric N_2_O from the study site using this system yielded isotope values of +6.8±0.7‰ for δ^15^N_bulk_, +44.1±1.7‰ for δ^18^O and +17.4±2.2‰ for SP_N2O_ (error reported as s.d. of *n*=6 samples). Reported values have been corrected for any size linearity effects on isotopic ratios (31/30, 45/44 and 46/44) by using a series of reference tank subsamples injected into He-purged 20 ml headspace vials using a gastight syringe. Precision for replicate analyses of this reference gas analysed as samples (that is, aliquots injected into sample vials and analysed via purge and trap) for δ^15^N is±0.3‰, for δ^18^O is ±0.4‰ and for SP_N2O_ is ±0.8‰. The Δ^17^O of N_2_O was calculated similar to that described above for NO_2_^−^. After extraction and cryotrapping, the N_2_O sample is thermally decomposed to N_2_ and O_2_ and chromatographically separated before measurement on the IRMS. Regular analyses of N_2_O converted from NO_3_^−^ isotope reference materials (USGS 35 and USGS 34) via the denitrifier method were made to normalize Δ^17^O values.

### Triple oxygen isotope tracing of N_2_O production

While the δ^18^O of most terrestrial O-bearing materials tightly co-varies with δ^17^O (along the ‘terrestrial fractionation line'), atmospheric NO_3_^−^, stemming from reactions involving stratospheric ozone, contains a large relative excess of ^17^O giving rise to a composition falling above the terrestrial fractionation line^79^ (with the magnitude of this anomaly expressed as Δ^17^O; see equation 2). Since kinetic isotope effects lead to mass dependent changes in δ^17^O values approximately half as large as in δ^18^O, the Δ^17^O remains unchanged^80^,^81^,^82^. Therefore N_2_O produced from this NO_3_^−^ (whether by bDNF, fDNF or cDNF) will retain its Δ^17^O, despite any kinetic isotope fractionation. Changes in Δ^17^O of an O-bearing N pool only occur through production incorporating O atoms having Δ^17^O ∼0‰ (O_2_ (Δ^17^O=−0.3‰) or H_2_O (Δ^17^O=0‰) incorporated during AMO), thus, decreasing the Δ^17^O of the standing pool towards ∼0‰. To the degree that NO_2_^−^ derives from NO_3_^−^ reduction (and hence carries its Δ^17^O value), isotope equilibration between NO_2_^−^ and water O may also ‘erase' a non-zero Δ^17^O signal. Thus, in our incubations Δ^17^O_N2O_ provides independent quantification of the fraction of O atoms originally deriving from NO_3_^−^ (through a NO_2_^−^ intermediate):


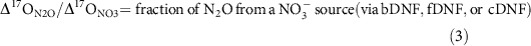


Normalizing for any potential equilibration of intermediate NO_2_^−^ with water, Δ^17^O_N2O_ can similarly be compared to Δ^17^O of the NO_2_^−^ pool:


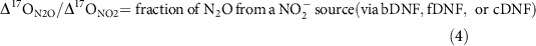


### Isotope mass balance approach

Isotope mass balance calculations were made for estimating the relative contribution of N_2_O production pathways in the sediment incubations (denitrification by bacteria (bDNF), by fungi (fDNF), or by chemodenitrification (cDNF), as well as combined production by ammonia oxidizing bacteria and archaea via NH_2_OH decomposition (AMO) or nitrifier denitrification (nDNF)). By combining four independent mass balance expressions (equations 5, 6, 9 and 11 below) we can solve for the contribution of four independent N_2_O production processes (here we describe consideration of fDNF (case 1), with cDNF being considered separately (case 2)). Equation 1 can be expressed in terms of the fractional contribution (f) of each production process to the total flux of N_2_O:


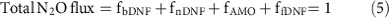


Equation 4, incorporating the Δ^17^O measurements, is used but neglecting cDNF for this case:


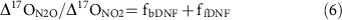


For measured Δδ^15^N values, equation 7 describes the mass balance contribution of each process to the cumulative steady-state flux (Δδ^15^N_meas_):


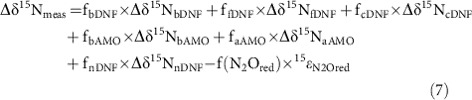


where again f refers to the fractional contribution of a given process, Δδ^15^N is equal to the steady-state difference (or offset) between δ^15^N of NO_3_^−^ and N_2_O, and where the endmember Δδ^15^N for given process is expressed based on measured steady-state δ^15^N values of NO_3_^−^, NO_2_^−^ or NH_4_^+^ and the isotope offsets for each process (Supplementary Table 3). For example, the difference between δ^15^N of reactant NH_3_ and N_2_O (^15^ɛ_NH3-N2O_) produced by bAMO has been estimated to be ∼3.7±3‰, with δ^15^N_N2Obulk_ depleted in ^15^N relative to the NH_3_ source^59^. To express this in terms of a Δδ^15^N value for the NO_3_^−^/N_2_O mass balance in equation 7, we also need to account for the steady-state difference between the δ^15^N of NO_3_^−^ and NH_4_^+^ such that:


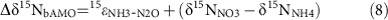


As the isotopic composition of N_2_O from bAMO and aAMO are very similar in the context of the isotope space evaluated here^56^,^57^,^58^, we choose to combine these terms into a single term (AMO), having the composition of bAMO (Supplementary Table 3). Together with the fact that we will treat cDNF separately, equation 7 thus simplifies to:


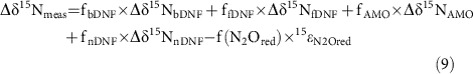


Similar to equation 7, the fractional contribution of each process to the measured SP_N2O_ of the effluent can be expressed as:


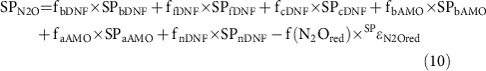


where f denotes the fractional contribution of a given process having a particular SP value, and where f(N_2_O_RED_) is equal to 1−(F_N2O_/(F_bDNF_+F_fDNF_+F_cDNF_+F_bAMO_+F_aAMO_+F_nDNF_)) and ^SP^ɛ_N2ORED_ is the kinetic isotope effect on SP for N_2_O reduction of −6‰ (refs 21, 22). As in equation 9, consideration of four processes simplifies equation 10 to:


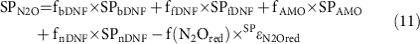


By combining equations 5, 6, 9 and 11—we can uniquely solve for the fractional contribution of four processes (bDNF, fDNF, AMO and nDNF) to the total observed N_2_O fluxes of the core incubations (Fig. 5; Supplementary Table 4). Isotope offsets (Δδ^15^N) and SP_N2O_ values for defining endmember compositions are given in Supplementary Table 3, as well as the expected Δ^17^O_N2O_/Δ^17^O_NO2_ values for the high nitrate incubations.

### Error propagation and sensitivity analysis

Error estimates for these mass balance calculations (Supplementary Tables 4,5 and 6) were calculated using a Monte Carlo error propagation approach in R (with 10,000 simulations), in which randomized Gaussian distributions of values were generated, as defined by their mean and s.d. given in Supplementary Table 3. This approach takes into account both the error associated with measurement of steady-state isotopic compositions (Supplementary Table 2; which implicitly incorporates both analytical error during instrument measurement, as well as natural variability during operation of the incubations) as well as error associated with the definition of endmembers (Supplementary Table 3).

Beyond these estimates of error, we also evaluated the sensitivity of specific endmember values to the calculated mass balance estimates—focusing on variations for endmember values having the least amount of certainty. First, the endmember values of Δδ^15^N for bDNF and fDNF (and cDNF) are prescribed to be low as an inferred consequence of diffusion-limited expression of intrinsic (enzyme level) isotope effects (Supplementary Table 3). For nDNF, we chose to use a Δδ^15^N value established by a study of nitrifiers under varying oxygen tension (Frame and Casciotti^59^), as nitrifiers will be growing at the sharp oxic/anoxic interface in our sediment core incubations. Whether their supply of NO_2_^−^ (as a substrate for nDNF) could be considered to be limited by diffusion is perhaps a matter of debate. However, we reasoned that nitrifiers will most generally be denitrifying the product NO_2_^−^ that they themselves are producing (for detoxification) and therefore would not be limited by diffusion of NO_2_^−^ from anoxic depths below. Further, the positive flux of NO_2_^−^ out of the sediments—also indicates that the diffusive supply of NO_2_^−^ should not have been limited (regardless of the source of the NO_2_^−^). Nevertheless, we discretely examined the impact that these assumptions make by decreasing the endmember Δδ^15^N value for nDNF from 56.9 to 28‰ and then to 14‰. Under these scenarios (assuming 10% N_2_O reduction, for example)—the average relative contribution of nDNF increases from 8 to 14 and 26%, respectively, though mostly at the expense of bDNF, which decreases on average from 45 to 38 and 25%, respectively. In comparison, these scenarios actually increase estimated contribution of fDNF from 36 to 43 and 56%, respectively (Supplementary Table 5).

Endmember SP values have been generally well established for bDNF, nDNF and bAMO through culture studies under a variety of growth conditions. SP for fungal DNF is admittedly less well studied, however, several studies have shown that SP values are universally elevated (often clustering ∼35–37‰). Recent studies^37^,^42^ observe that most N_2_O producing fungal cultures yielded SP values >30‰. Finally, even the purified N_2_O producing fungal enzyme (p450nor) has been shown to exhibit elevated SP values, albeit at somewhat lower values (15–29‰). Notably, our choice of +37‰ for the fDNF endmember is conservative in estimation of the relative contribution of fDNF. For example, decreasing this value to the mean reported by Maeda *et al*.^37^ of +30.3±4.8‰ (which notably also contained some questionably low values), results in an average of a 6% increase in the contribution of fDNF to N_2_O production, with the average contribution of 36% shifting up to an average of 41–42% (Supplementary Table 6).

Finally, we evaluated our assumption that the extracellular NO_2_^−^ pool could be disregarded as a reactant source for nDNF (that *in situ*, nDNF only occurred from NO_2_^−^ supplied via bAMO), by setting the prescribed Δ^17^O-N_2_O/Δ^17^O-NO_2_^−^ value for nDNF to a value of ‘1' instead of ‘0'—representing the most extreme case. Indeed, under this scenario—the average estimated contribution of bNDF and nDNF actually do not change by >∼1%, while bAMO is increased (from 12 to 19%) and fDNF is decreased (from 36 to 28%) (Supplementary Table 6).

### Fungal genetic sequencing

Sediment samples for fungal sequence analysis were collected and stored frozen at −80 °C. Genomic DNA from marine sediment samples was extracted using a bead beating protocol according to the manufacturer's instruction (Mo Bio, Carlsbad, CA). ITS region sequences were amplified using the fungal ITS primer pair F (ITS5): GGAAGTAAAAGTCGTAACAAGG and R (ITS4): TCCTCCGCTTATTGATATGC generating fragments of ∼600 bps in length. PCR products were cloned using Zero Blunt TOPO PCR Cloning (Thermo Fisher, Carlsbad, CA). After a ligate buffer exchange the plasmid was transferred into TOP10 electrocompetent cells. Cells were plated and grown on LB agar containing kanamycin. Single colonies were recovered from each plate and amplified using M13F&R primers. The products were sequenced by Sanger method (EtonBio, San Diego, CA). Sequences were analysed using BLAST. Taxonomy was assigned for fungal sequences (Supplementary Figure S3) by comparison against untrimmed ITS in the UNITE database (01/08/2015 version), using QIIME v1.91. Sequences were assigned only if the database match had a similarity of at least 90% and maximum e-value of 0.001.

### Data availability

The data sets generated during this study are available by request from corresponding author.

## Additional information

**How to cite this article:** Wankel, S. D. *et al*. Evidence for fungal and chemodenitrification based N_2_O flux from nitrogen impacted coastal sediments. *Nat. Commun.* **8**, 15595 doi: 10.1038/ncomms15595 (2017).

**Publisher's note:** Springer Nature remains neutral with regard to jurisdictional claims in published maps and institutional affiliations.

## Supplementary Material

Supplementary Information Supplementary Figures and Supplementary Tables

## Figures and Tables

**Figure 1:**
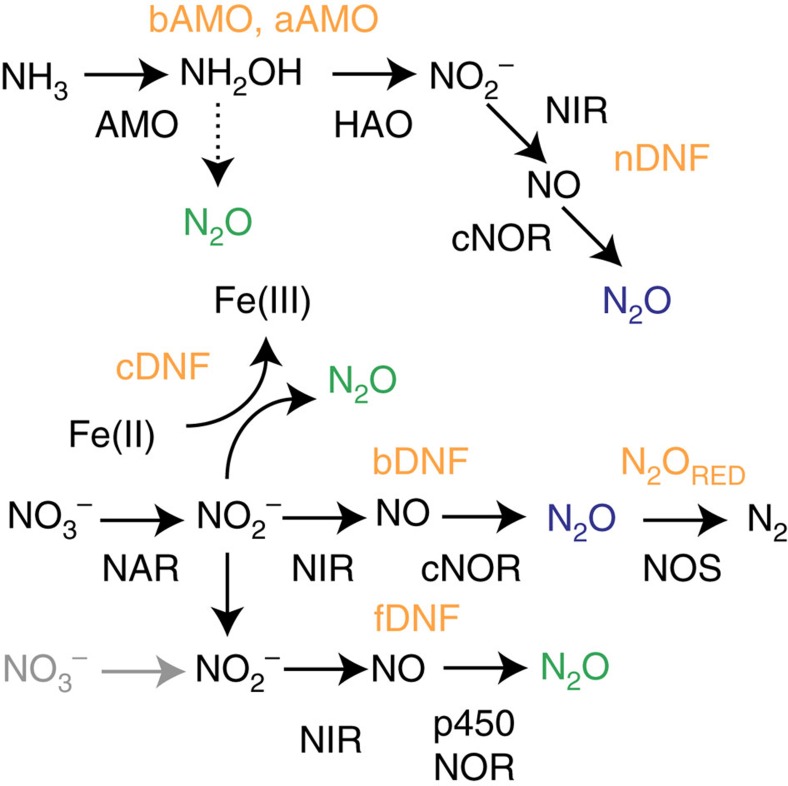
Schematic representation of N_2_O production pathways considered in this study. N_2_O can form during decomposition/reaction of intermediate hydroxylamine (NH_2_OH) produced during ammonia oxidation by bacteria (bAMO) or archaea (aAMO). Some of these nitrifying organisms may also produce N_2_O during reduction of product nitrite (NO_2_^−^), known as nitrifier-denitrification (nDNF). N_2_O may also be produced during reduction of nitrate and/or nitrite by denitrification catalysed by bacteria (bDNF), fungi (fDNF) and/or by chemical reaction with Fe(II) or ‘chemodenitrification' (cDNF). Finally, N_2_O can also be reductively consumed by denitrifying bacteria (N_2_O_RED_). N_2_O produced having low or negative site preference values (−10 to 0‰) are indicated in blue, while those have high site preference values (>15‰) are indicated in green. Enzymes are indicated as ammonia monooxygenase (AMO), hydroxylamine oxidoreductase in bacterial nitrification (HAO), nitrate reductase (NAR), nitrite reductase (NIR), bacterial nitric oxide reductase (cNOR), nitrous oxide reductase (NOS) and fungal nitric oxide reductase (p450NOR). The detailed biochemical pathway of nitrite production by archaeal ammonia oxidation remains unclear.

**Table 1:** Steady state mass fluxes of measured nitrogen species.

	**NO**_**3**_^−^ **flux (mmol** **m**^−**2**^ **d**^−**1**^**)**	**NO**_**2**_^−^ **flux (mmol** **m**^−**2**^ **d**^−**1**^**)**	**NH**_**4**_^**+**^ **flux (mmol** **m**^−**2**^ **d**^−**1**^**)**	**N**_**2**_**O flux (μmol** **m**^−**2**^ **d**^−**1**^**)**
*Mean by treatment*				
Low NO_3_^−^	−4.7±1.1	0.7±0.3	7.1±1.7	17.8±6.5
Low O_2_, Low NO_3_^−^	−4.1±1.0	0.7±0.3	8.3±1.2	5.9±6.6
High NO_3_^−^	−10.4±1.4	1.3±0.2	8.6±1.4	49.0±18.5
Low O_2_, High NO_3_^−^	−17.4±2.1	2.4±0.7	7.1±1.4	51.1±23.1
*Mean by site*				
Mud (MD)	−9.4±1.2	1.1±0.4	10.7±1.4	31.9±17.0
Mixed (MX)	−7.6±1.8	1.2±0.2	7.9±1.3	31.1±12.3
Sand (SD)	−10.5±1.3	1.5±0.6	4.7±1.5	29.9±11.7

Mean values are shown grouped either by treatment type or by site. Negative values refer to uptake by the sediments.

**Table 2:** Steady state isotopic composition of measured nitrogen species.

	**NO**_**3**_^−^		**NO**_**2**_^−^		**TRN**	**N**_**2**_**O**		
	δ^**15**^**N**	Δ^**17**^**O**	δ^**15**^**N**	Δ^**17**^**O**	δ^**15**^**N**	δ^**15**^**N**	Δ^**17**^**O**	**SP**
*By treatment*								
Low O_2_ (LO)	14.0±1.0	^	6.6±2.2	^	11.9±1.1	13.7±1.7	^	7.2±3.4
Low O_2_, Low NO_3_^−^ (LOLN)	14.0±0.6	^	7.7±2.7	^	11.6±1.9	14.5±2.0	^	6.2±3.2
High NO_3_^−^ (HN)	4.6±0.2	14.7±0.7	−1.2±1.8	8.5±2.4	11.9±1.1	0.0±0.6	6.5±1.2	16.2±5.0
Low O_2_, High NO_3_^−^ (LOHN)	5.3±0.5	14.7±0.6	−1.5±1.9	9.2±3.2	12.9±6.8	−0.7±1.1	5.4±1.3	12.9±2.5
*By site*								
Mud (MD)	9.2±0.5	15.2±0.7	1.6±1.9	5.4±2.5	12.0±1.7*	6.4±1.2	5.3±1.5	12.5±4.3
Mixed (MX)	9.4±0.6	14.5±0.6	2.8±1.7	10.0±2.3	13.5±1.5	5.8±0.9	6.6±1.2	6.4±3.5
Sand (SD)	9.9±0.6	14.5±0.6	4.3±2.8	11.1±3.5	11.1±2.6	8.3±1.9	5.9±1.1	13.0±3.0
*Inflow*								
Low O_2_ (LO)	12.7±1.2	0.0±0.6	ND	ND	^	5.7±0.6	^	17.4±1.0
Low O_2_, Low NO_3_^−^ (LOLN)	11.7±0.5	0.0±0.6	ND	ND	^	5.7±0.6	^	17.4±1.0
High NO_3_^−^ (HN)	4.3±0.3	15.3±0.7	ND	ND	^	5.7±0.6	^	17.4±1.0
Low O_2_, High NO_3_^−^ (LOHN)	3.9±0.3	15.0±0.5	ND	ND	^	5.7±0.6	^	17.4±1.0

Mean values are shown for either treatment type or by site. Values are also shown for the water used as inflow. All values are in units of permil (‰) with error indicated as ±1 s.d. based on all measurements conducted during steady-state conditions. TRN refers to total reduced nitrogen equal to the combined NH_4_^+^ and dissolved organic nitrogen pool. ND=not detected. Δ^17^O was only measured in effluent of experiments in which NO_3_^−^ was amended. The symbol (^) indicates no measurements were made.
