# Supplementary material for: Evidence for fungal and chemodenitrification based N2O flux from nitrogen impacted coastal sediments
Source: Nat Commun. 2017 Jun 5;8:15595. doi: 10.1038/ncomms15595 (PMC5465357; doi:10.1038/ncomms15595)
Supplement: Supplementary Information — Supplementary Figures and Supplementary Tables [file ncomms15595-s1.pdf]

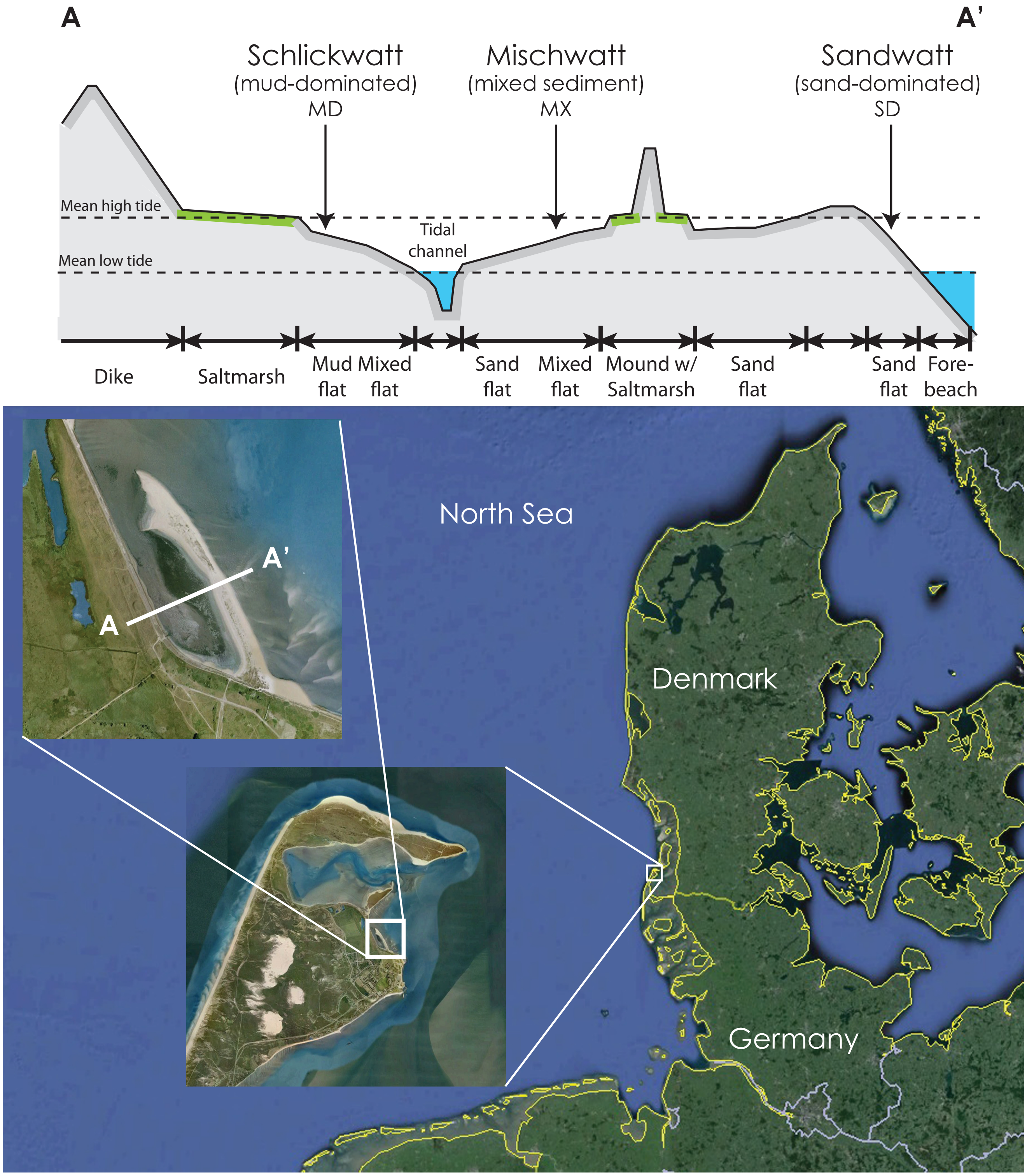

Supplemenatry Figure 1. Map illustrating the sampling locations on the island of Sylt, Germany. Three sites were located on a seaward transect (A to A') just north of the Alfred Wegener Institute for Wadden Sea Research in List. Sites were ~100m apart and chosen based on qualitative differences in sediment grain size and location characteristics. The 'Schlickwatt (MD)' and 'Mischwatt (MX)' sites were located in a small lagoon (Tonenleger Bucht), representing a more protected and low-energy depositional environment, while the 'Sandwatt (SD)' site was located on a beach with open exposure to wind and waves. MX was located approximately mid-way between the MD and SD. While fine-grained sediments of sites MD and MX appeared dark and reducing within several mm below the water interface, sediments from site SD contained a larger sand fraction and appeared qualitatively coarser grained and less reducing. Map was produced using Google Earth (ver 7.1.8.3036; 32-bit).

Supplementary Figure 2. Steady-state fluxes of nitrogen species for all 24 core incubations (three sites, two cores per site, four treatments). Fluxes were calculated as the product of the mean concentration differences between inflow and outflow water and measured flow rates, normalized to surface area of sediment water interface. Error bar represents one standard deviation. Note scale change for N_2O flux.

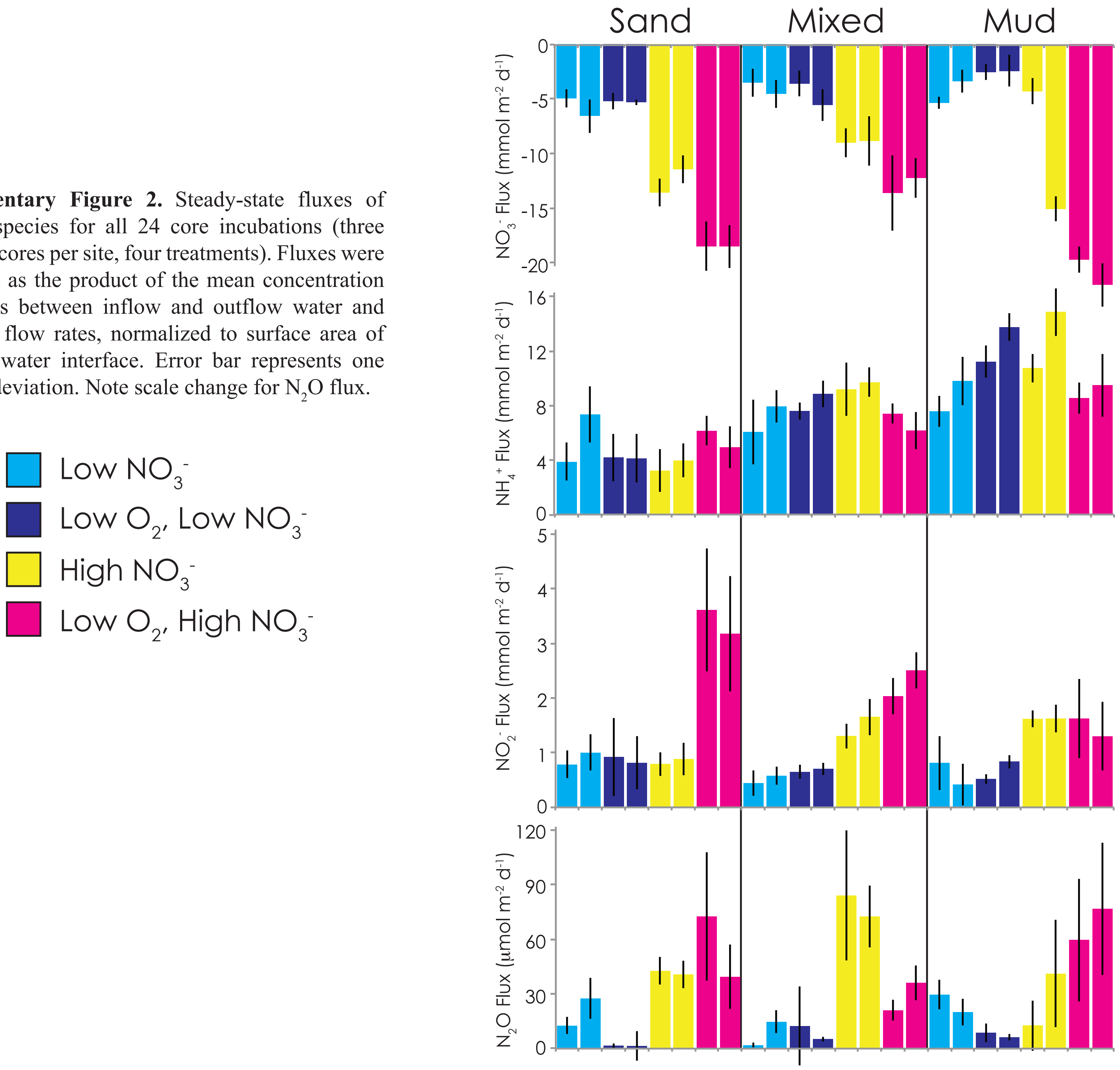

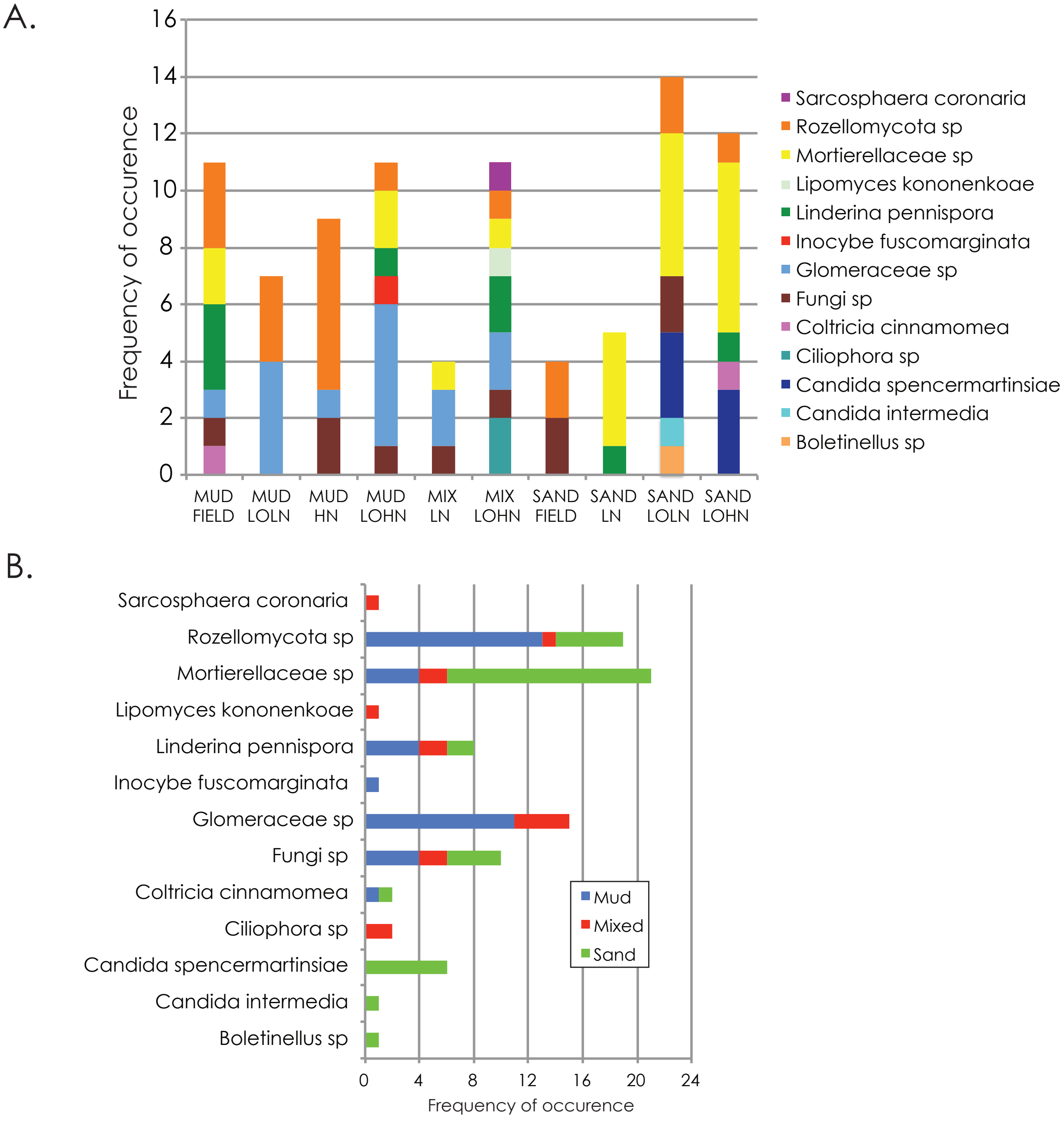

Supplementary Figure 3. Identity of fungal sequences retrieved from the upper 1cm of sediment cores. Taxonomy was assigned for fungal sequences using BLAST and by comparing it against untrimmed ITS in the UNITE database (01/08/2015 version), using QIIME v1.91. Sequences were assigned only if the database match had a similarity of at least 90% and maximum e-value of 0.001.

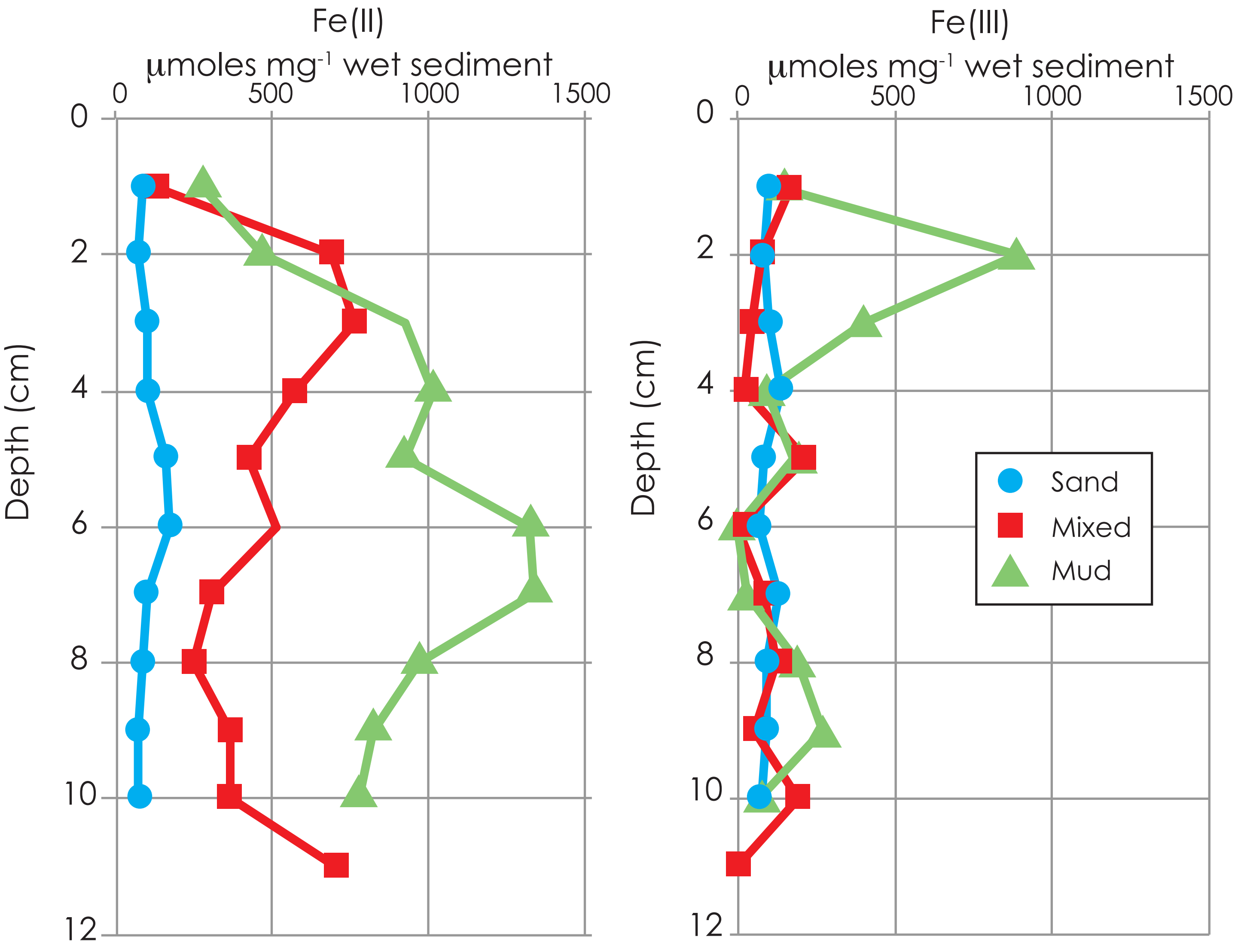

Supplementary Figure 4. Profiles from sediment cores at each site sampled immediately after collection of a) total HCl extractable Fe(II) and b) the poorly crystalline Fe(III) fraction following the procedures described in (Lovley et al, 1987), as modified by Kostka and colleagues (1994).

Supplementary Table 1. Steady-state mass fluxes of measured N bearing species. Negative values refer to uptake by the sediments.

Conditions	Site	Replicate	NO_3^- Flux (mmol m^{-2} d^{-1})			NO_2^- Flux (mmol m^{-2} d^{-1})			NH_4^+ Flux (mmol m^{-2} d^{-1})			N_2O Flux (μmol m^{-2} d^{-1})		
Low NO3	MD	A	-5.4	±	0.5	0.8	±	0.5	7.6	±	1.1	29.7	±	8.1
Low NO3	MD	B	-3.4	±	1.0	0.4	±	0.4	9.8	±	1.8	20.0	±	7.3
Low NO3	SD	A	-5.0	±	0.8	0.8	±	0.2	3.9	±	1.4	12.6	±	4.7
Low NO3	SD	B	-6.6	±	1.5	1.0	±	0.3	7.3	±	2.1	27.6	±	11.2
Low NO3	MX	A	-3.5	±	1.3	0.4	±	0.2	6.0	±	2.4	1.9	±	1.2
Low NO3	MX	B	-4.6	±	1.3	0.6	±	0.2	7.9	±	1.2	14.7	±	6.3
Low O2, Low NO3	MD	A	-2.6	±	0.7	0.5	±	0.1	11.2	±	1.2	8.8	±	5.2
Low O2, Low NO3	MD	B	-2.5	±	1.5	0.8	±	0.1	13.7	±	1.0	6.2	±	1.6
Low O2, Low NO3	SD	A	-5.2	±	0.7	0.9	±	0.7	4.2	±	1.7	1.6	±	1.3
Low O2, Low NO3	SD	B	-5.3	±	0.3	0.8	±	0.5	4.1	±	1.8	1.3	±	8.2
Low O2, Low NO3	MX	A	-3.6	±	1.2	0.6	±	0.1	7.6	±	0.6	12.4	±	21.7
Low O2, Low NO3	MX	B	-5.6	±	1.5	0.7	±	0.1	8.8	±	1.0	5.3	±	1.3
High NO3	MD	A	-4.3	±	1.2	1.6	±	0.2	10.7	±	1.1	12.7	±	13.9
High NO3	MD	B	-15.1	±	1.1	1.6	±	0.3	14.9	±	1.8	41.3	±	29.4
High NO3	SD	A	-13.6	±	1.2	0.8	±	0.2	3.2	±	1.6	42.6	±	7.6
High NO3	SD	B	-11.4	±	1.3	0.9	±	0.3	4.0	±	1.2	40.8	±	7.5
High NO3	MX	A	-9.0	±	1.4	1.3	±	0.2	9.2	±	2.0	84.2	±	35.7
High NO3	MX	B	-8.9	±	2.3	1.7	±	0.3	9.7	±	1.1	72.7	±	17.1
Low O2, High NO3	MD	A	-19.7	±	1.2	1.6	±	0.7	8.5	±	1.1	59.8	±	33.8
Low O2, High NO3	MD	B	-22.0	±	2.0	1.3	±	0.6	9.5	±	2.3	76.9	±	36.6
Low O2, High NO3	SD	A	-18.5	±	2.3	3.6	±	1.1	6.1	±	1.1	72.7	±	35.3
Low O2, High NO3	SD	B	-18.5	±	2.0	3.2	±	1.1	4.9	±	1.5	39.5	±	17.7
Low O2, High NO3	MX	A	-13.6	±	3.4	2.0	±	0.3	7.4	±	0.7	21.1	±	5.6
Low O2, High NO3	MX	B	-12.3	±	1.8	2.5	±	0.3	6.1	±	1.4	36.3	±	9.7

Supplementary Table 2. Steady-state isotopic composition of measured N bearing species

			NO_3^-		NO_2^-		TRN (NH_4^+ + DON)#	N_2O		
**Conditions**	**Site**	**Replicate**	**$\delta^{15}N$ (‰)**	**$\Delta^{17}O$ (‰)**	**$\delta^{15}N$ (‰)**	**$\Delta^{17}O$ (‰)**	**$\delta^{15}N$ (‰)**	**$\delta^{15}N$ (‰)**	**$\Delta^{17}O$ (‰)**	**Site Preference (‰)**
Low NO3	MD	A	14.1 ± 0.6	^ ^	7.4 ± 2.5	^ ^	12.0* ± 1.7*	14.9 ± 2.3	^ ^	8.4 ± 4.4
Low NO3	MD	B	13.0 ± 1.3	^ ^	5.1 ± 2.0	^ ^	12.0* ± 1.7*	15.4 ± 2.0	^ ^	21.3 ± 6.2
Low NO3	SD	A	15.1 ± 1.3	^ ^	9.2 ± 3.3	^ ^	10.9 ± 1.4	14.1 ± 1.9	^ ^	8.4 ± 0.7
Low NO3	SD	B	14.8 ± 0.9	^ ^	7.5 ± 3.3	^ ^	10.5 ± 0.8	16.8 ± 1.6	^ ^	1.6 ± 2.1
Low NO3	MX	A	13.1 ± 0.6	^ ^	5.5 ± 1.1	^ ^	13.1 ± 0.9	9.9 ± 1.3	^ ^	1.6 ± 3.6
Low NO3	MX	B	14.0 ± 1.4	^ ^	5.0 ± 1.2	^ ^	13.1 ± 1.3	11.0 ± 1.1	^ ^	1.7 ± 3.3
Low O2, Low NO3	MD	A	12.5 ± 0.3	^ ^	3.4 ± 2.1	^ ^	12.0* ± 1.7*	11.6 ± 1.1	^ ^	4.0 ± 3.3
Low O2, Low NO3	MD	B	14.4 ± 0.9	^ ^	3.9 ± 3.0	^ ^	12.0* ± 1.7*	16.0 ± 1.9	^ ^	4.1 ± 2.3
Low O2, Low NO3	SD	A	15.4 ± 0.3	^ ^	10.2 ± 2.3	^ ^	9.8 ± 1.8	18.1 ± 2.3	^ ^	-2.6 ± 1.9
Low O2, Low NO3	SD	B	14.0 ± 0.6	^ ^	10.7 ± 5.2	^ ^	9.6 ± 1.3	15.7 ± 4.6	^ ^	17.2 ± 4.5
Low O2, Low NO3	MX	A	13.0 ± 0.6	^ ^	8.7 ± 1.6	^ ^	15.5 ± 2.7	12.1 ± 1.2	^ ^	5.6 ± 4.6
Low O2, Low NO3	MX	B	14.8 ± 0.7	^ ^	9.1 ± 2.1	^ ^	12.0* ± 1.7*	13.4 ± 0.7	^ ^	8.9 ± 2.3
High NO3	MD	A	4.6 ± 0.2	14.4 ± 1.2	-0.4 ± 1.0	5.6 ± 3.0	12.0* ± 1.7*	-3.6 ± 0.2	5.0 ± 1.5	25.4 ± 9.8
High NO3	MD	B	5.1 ± 0.3	14.6 ± 0.5	-2.0 ± 1.9	2.8 ± 2.2	12.0* ± 1.7*	-3.6 ± 1.2	2.7 ± 1.5	12.1 ± 2.8
High NO3	SD	A	4.3 ± 0.2	14.7 ± 0.6	-0.6 ± 3.3	10.6 ± 4.9	11.3 ± 1.5	2.4 ± 0.3	8.4 ± 1.3	32.6 ± 5.5
High NO3	SD	B	4.4 ± 0.2	14.7 ± 0.6	-2.4 ± 1.8	10.1 ± 0.9	10.8 ± 0.7	2.0 ± 0.8	8.6 ± 1.5	20.4 ± 3.0
High NO3	MX	A	4.7 ± 0.2	15.1 ± 0.4	-1.1 ± 0.9	8.9 ± 3.0	12.7 ± 1.0	1.9 ± 0.3	6.8 ± 0.8	0.2 ± 3.8
High NO3	MX	B	4.8 ± 0.1	14.8 ± 0.7	-0.6 ± 1.6	13.0 ± 0.1	13.0 ± 1.3	1.0 ± 0.6	7.6 ± 0.8	6.3 ± 5.2
Low O2, High NO3	MD	A	4.9 ± 0.2	14.4 ± 0.5	-1.4 ± 1.4	6.4 ± 1.0	12.0* ± 1.7*	0.5 ± 0.6	6.5 ± 1.9	13.6 ± 2.7
Low O2, High NO3	MD	B	5.0 ± 0.3	17.2 ± 0.7	-3.2 ± 1.1	6.9 ± 3.8	12.0* ± 1.7*	0.1 ± 0.2	7.2 ± 1.0	11.0 ± 2.5
Low O2, High NO3	SD	A	5.9 ± 1.1	14.1 ± 0.9	-0.9 ± 1.7	9.4 ± 8.1	13.6 ± 6.4	-1.4 ± 1.7	4.6 ± 0.5	* ± *
Low O2, High NO3	SD	B	5.4 ± 0.3	14.4 ± 0.3	0.7 ± 2.0	14.3 ± 0.2	12.2 ± 7.2	-0.8 ± 1.9	1.9 ± 1.0	* ± *
Low O2, High NO3	MX	A	5.0 ± 0.3	14.3 ± 0.6	-1.0 ± 1.9	9.6 ± 1.8	12.0* ± 1.7*	-1.1 ± 0.9	5.6 ± 2.3	12.3 ± 3.3
Low O2, High NO3	MX	B	5.6 ± 0.7	13.9 ± 0.7	-2.9 ± 3.5	8.5 ± 4.2	12.0* ± 1.7*	-1.5 ± 1.1	6.4 ± 1.1	14.6 ± 1.6
**Means by Treatment**										
**Low NO3**			14.0 ± 1.0	^ ^	6.6 ± 2.2	^ ^	11.9 ± 1.1	13.7 ± 1.7	^ ^	7.2 ± 3.4
**Low O2, Low NO3**			14.0 ± 0.6	^ ^	7.7 ± 2.7	^ ^	11.6 ± 1.9	14.5 ± 2.0	^ ^	6.2 ± 3.2
**High NO3**			4.6 ± 0.2	14.7 ± 0.7	-1.2 ± 1.8	8.5 ± 2.4	11.9 ± 1.1	0.0 ± 0.6	6.5 ± 1.2	16.2 ± 5.0
**Low O2, High NO3**			5.3 ± 0.5	14.7 ± 0.6	-1.5 ± 1.9	9.2 ± 3.2	12.9 ± 6.8	-0.7 ± 1.1	5.4 ± 1.3	12.9 ± 2.5
**Means by Site**										
**Mud (MD)**			9.2 ± 0.5	15.2 ± 0.7	1.6 ± 1.9	5.4 ± 2.5	12.0* ± 1.7*	6.4 ± 1.2	5.3 ± 1.5	12.5 ± 4.3
**Mixed (MX)**			9.4 ± 0.6	14.5 ± 0.6	2.8 ± 1.7	10.0 ± 2.3	13.5 ± 1.5	5.8 ± 0.9	6.6 ± 1.2	6.4 ± 3.5
**Sand (SD)**			9.9 ± 0.6	14.5 ± 0.6	4.3 ± 2.8	11.1 ± 3.5	11.1 ± 2.6	8.3 ± 1.9	5.9 ± 1.1	13.0 ± 3.0
**Inflow**										
**Low NO3**			12.7 ± 1.2	0.0 ± 0.6	- -	- -	- -	5.7 ± 0.6	^ ^	17.4 ± 1.0
**Low O2, Low NO3**			11.7 ± 0.5	0.0 ± 0.6	- -	- -	- -	5.7 ± 0.6	^ ^	17.4 ± 1.0
**High NO3**			4.3 ± 0.3	15.3 ± 0.7	- -	- -	- -	5.7 ± 0.6	^ ^	17.4 ± 1.0
**Low O2, High NO3**			3.9 ± 0.3	15.0 ± 0.5	- -	- -	- -	5.7 ± 0.6	^ ^	17.4 ± 1.0

#Total reduced nitrogen isotopes were estimated as the mass balance difference between the $\delta^{15}N$ of total dissolved nitrogen and the $\delta^{15}N$ of nitrate and nitrite.

*Total reduced nitrogen isotopes were measured in only a subset of samples, the average value and standard deviation of which was used for mass balance calculations.

^not measured

Supplementary Table 3. Isotope offsets, site preference values and expected $\Delta^{17}O$ incorporation used for defining endmember compositions for mass balance calculations.

Process	Site Preference (SP)			$\Delta\delta^{15}N$ (‰)			$\Delta^{17}O_{N2O}$ / $\Delta^{17}O_{NO2}$	References
**Bacterial Denitrification (bDNF)**	-4.9	±	1.8					Toyoda et al., 2005; Yamazaki et al., 2013
$\delta^{15}N_{NO3}$ - $\delta^{15}N_{N2O}$					0‰*			Brandes and Devol, 1997
$\Delta^{17}O_{N2O}$ / $\Delta^{17}O_{NO2}$							1	this study
**Fungal Denitrification (fDNF)**	37	±	2.7					Sutka et al., 2008; Rohe et al., 2014
$\delta^{15}N_{NO3}$ - $\delta^{15}N_{N2O}$				6.6*	±	3		Sutka et al., 2006
$\Delta^{17}O_{N2O}$ / $\Delta^{17}O_{NO2}$							1	this study
**Chemodenitrification (cDNF)**	16	±	5					Jones et al., 2014; Buchwald et al., 2016
$\delta^{15}N_{NO2}$ - $\delta^{15}N_{N2O}$				4*	±	4		Jones et al., 2014; Buchwald et al., 2016
$\Delta^{17}O_{N2O}$ / $\Delta^{17}O_{NO2}$							1	this study
**Bacterial Ammonia Oxidation (bAMO)#**	36.3	±	2.4					Frame and Casciotti, 2010
$\delta^{15}N_{NH3}$ - $\delta^{15}N_{N2O}$				3.7	±	3		Frame and Casciotti, 2010
$\Delta^{17}O_{N2O}$ / $\Delta^{17}O_{NO2}$							0	this study
**Archael Ammonia Oxidation (aAMO)#**	32.4	±	5.6					Santoro et al., 2011; Löscher et al., 2012
$\delta^{15}N_{NH3}$ - $\delta^{15}N_{N2O}$				-6.2	±	3		Santoro et al., 2011
$\Delta^{17}O_{N2O}$ / $\Delta^{17}O_{NO2}$							0	this study
**Nitrifier Denitrification (nDNF)**	-10.7	±	2.9					Frame and Casciotti, 2010
$\delta^{15}N_{NH3}$ - $\delta^{15}N_{N2O}$				56.9	±	3.8		Frame and Casciotti, 2010
$\Delta^{17}O_{N2O}$ / $\Delta^{17}O_{NO2}$							0	this study

*values assumed for diffusion-limited conditions

#values for bAMO were assumed to include those of aAMO for mass balance calculations, as exact endmember values for aAMO remain undetermined

Supplementary Table 4. Fractional contribution of N_2O production calculated by isotope mass balance. Error estimated by Monte Carlo simulation. See text for details.
Case 1 explicitly examines the contribution of fungal denitrification (ignoring chemodenitrification), while Case 2 considers the contribution of chemodenitrification in lieu of fungal denitrification.
See Supplemental Tables 5 and 6 for alternative scenarios and sensitivity analyses.

Case 1 (base case)

			**Bacterial Denitrification**						**Nitrifier Denitrification**						**Bacterial Ammonia Oxidation**						**Fungal Denitrification**					
**Conditions**	**Site**	**Replicate**	at 10% N_2O reduction			at 90% N_2O reduction			at 10% N_2O reduction			at 90% N_2O reduction			at 10% N_2O reduction			at 90% N_2O reduction			at 10% N_2O reduction			at 90% N_2O reduction		
High NO3	Mud	A	0.19	±	0.19	0.19	±	0.19	0.09	±	0.05	0.18	±	0.05	0.01	±	0.12	-0.08	±	0.12	0.71	±	0.22	0.71	±	0.22
High NO3	Mud	B	0.46	±	0.09	0.45	±	0.10	0.13	±	0.04	0.22	±	0.04	-0.03	±	0.18	-0.12	±	0.18	0.44	±	0.19	0.44	±	0.19
High NO3	Sand	A	0.15	±	0.12	0.14	±	0.11	-0.02	±	0.04	0.07	±	0.04	0.23	±	0.10	0.14	±	0.10	0.66	±	0.15	0.66	±	0.15
High NO3	Sand	B	0.39	±	0.07	0.39	±	0.07	0.01	±	0.02	0.10	±	0.02	0.19	±	0.04	0.10	±	0.04	0.41	±	0.07	0.41	±	0.07
High NO3	Mixed	A	0.78	±	0.08	0.78	±	0.08	0.09	±	0.02	0.18	±	0.02	0.11	±	0.06	0.02	±	0.06	0.01	±	0.09	0.02	±	0.10
High NO3	Mixed	B	0.60	±	0.10	0.59	±	0.10	0.13	±	0.03	0.22	±	0.03	0.37	±	0.03	0.28	±	0.03	-0.09	±	0.11	-0.09	±	0.11
Low O2, High NO3	Mud	A	0.53	±	0.07	0.53	±	0.07	0.04	±	0.03	0.13	±	0.03	-0.04	±	0.07	-0.13	±	0.07	0.47	±	0.09	0.47	±	0.09
Low O2, High NO3	Mud	B	0.57	±	0.07	0.57	±	0.07	0.05	±	0.04	0.14	±	0.04	-0.05	±	0.11	-0.14	±	0.11	0.42	±	0.12	0.43	±	0.12
Low O2, High NO3	Mixed	A	0.45	±	0.07	0.45	±	0.07	0.13	±	0.03	0.22	±	0.03	0.27	±	0.09	0.18	±	0.09	0.15	±	0.11	0.15	±	0.11
Low O2, High NO3	Mixed	B	0.42	±	0.05	0.41	±	0.05	0.11	±	0.03	0.20	±	0.03	0.09	±	0.10	0.00	±	0.10	0.38	±	0.11	0.39	±	0.11
		**Average**	**0.45**		**0.09**	**0.45**		**0.09**	**0.08**		**0.03**	**0.17**		**0.03**	**0.12**		**0.09**	**0.03**		**0.09**	**0.36**		**0.12**	**0.36**		**0.13**

Case 2 (base case)

			**Bacterial Denitrification**						**Nitrifier Denitrification**						**Bacterial Ammonia Oxidation**						**Chemodenitrification**					
**Conditions**	**Site**	**Replicate**	at 10% N_2O reduction			at 90% N_2O reduction			at 10% N_2O reduction			at 90% N_2O reduction			at 10% N_2O reduction			at 90% N_2O reduction			at 10% N_2O reduction			at 90% N_2O reduction		
High NO3	Mud	A	-0.46	±	0.46	-0.48	±	0.46	0.08	±	0.09	0.17	±	0.09	0.03	±	0.14	-0.06	±	0.14	1.37	±	0.50	1.39	±	0.50
High NO3	Mud	B	0.06	±	0.26	0.05	±	0.26	0.13	±	0.07	0.22	±	0.07	-0.02	±	0.18	-0.11	±	0.18	0.85	±	0.41	0.86	±	0.41
High NO3	Sand	A	-0.47	±	0.34	-0.48	±	0.34	-0.04	±	0.08	0.05	±	0.08	0.24	±	0.12	0.15	±	0.12	1.27	±	0.38	1.29	±	0.38
High NO3	Sand	B	0.01	±	0.20	0.00	±	0.20	0.00	±	0.05	0.09	±	0.05	0.20	±	0.06	0.11	±	0.06	0.79	±	0.21	0.81	±	0.22
High NO3	Mixed	A	0.77	±	0.16	0.76	±	0.15	0.09	±	0.02	0.18	±	0.02	0.11	±	0.06	0.02	±	0.06	0.02	±	0.19	0.03	±	0.18
High NO3	Mixed	B	0.68	±	0.21	0.67	±	0.21	0.13	±	0.03	0.22	±	0.03	0.37	±	0.04	0.28	±	0.04	-0.18	±	0.21	-0.17	±	0.21
Low O2, High NO3	Mud	A	0.09	±	0.22	0.08	±	0.22	0.03	±	0.06	0.12	±	0.06	-0.02	±	0.08	-0.11	±	0.09	0.91	±	0.25	0.92	±	0.25
Low O2, High NO3	Mud	B	0.18	±	0.21	0.17	±	0.22	0.04	±	0.06	0.14	±	0.06	-0.04	±	0.12	-0.13	±	0.12	0.82	±	0.29	0.83	±	0.29
Low O2, High NO3	Mixed	A	0.31	±	0.16	0.30	±	0.16	0.13	±	0.04	0.22	±	0.04	0.27	±	0.08	0.18	±	0.09	0.29	±	0.21	0.31	±	0.22
Low O2, High NO3	Mixed	B	0.07	±	0.17	0.06	±	0.18	0.11	±	0.05	0.19	±	0.05	0.10	±	0.11	0.02	±	0.10	0.73	±	0.25	0.74	±	0.25
		**Average**	**0.12**		**0.24**	**0.11**		**0.24**	**0.07**		**0.05**	**0.16**		**0.05**	**0.13**		**0.10**	**0.04**		**0.10**	**0.69**		**0.29**	**0.70**		**0.29**

Supplementary Table 5. Sensitivity analysis of fractional estimates of N_2O production calculated by isotope mass balance. Error estimated by Monte Carlo simulation. See text for details. Compare to table S4 (base case) for relative magnitude of changes.

Consideration of a lower offset value for nDNF ($\Delta\delta^{15}N$ = 28‰)

			Bacterial Denitrification						Nitrifier Denitrification						Bacterial Ammonia Oxidation						Fungal Denitrification					
Conditions	Site	Replicate	at 10% N_2O reduction			at 90% N_2O reduction			at 10% N_2O reduction			at 90% N_2O reduction			at 10% N_2O reduction			at 90% N_2O reduction			at 10% N_2O reduction			at 90% N_2O reduction		
High NO3	Mud	A	0.10	±	0.19	0.00	±	0.20	0.17	±	0.10	0.34	±	0.11	-0.07	±	0.12	-0.24	±	0.13	0.80	±	0.20	0.90	±	0.21
High NO3	Mud	B	0.32	±	0.12	0.23	±	0.14	0.25	±	0.09	0.42	±	0.10	-0.14	±	0.16	-0.32	±	0.17	0.57	±	0.17	0.67	±	0.18
High NO3	Sand	A	0.17	±	0.12	0.07	±	0.12	-0.05	±	0.07	0.12	±	0.07	0.25	±	0.10	0.08	±	0.10	0.63	±	0.14	0.73	±	0.14
High NO3	Sand	B	0.38	±	0.07	0.28	±	0.08	0.02	±	0.05	0.19	±	0.05	0.18	±	0.06	0.01	±	0.06	0.42	±	0.08	0.52	±	0.08
High NO3	Mixed	A	0.69	±	0.08	0.60	±	0.09	0.17	±	0.04	0.34	±	0.06	0.03	±	0.05	-0.15	±	0.07	0.11	±	0.08	0.21	±	0.09
High NO3	Mixed	B	0.46	±	0.10	0.36	±	0.11	0.24	±	0.05	0.41	±	0.06	0.26	±	0.05	0.08	±	0.06	0.04	±	0.10	0.14	±	0.11
Low O2, High NO3	Mud	A	0.49	±	0.08	0.39	±	0.10	0.07	±	0.06	0.24	±	0.07	-0.07	±	0.08	-0.24	±	0.09	0.51	±	0.09	0.60	±	0.11
Low O2, High NO3	Mud	B	0.52	±	0.10	0.42	±	0.11	0.10	±	0.08	0.27	±	0.09	-0.10	±	0.11	-0.27	±	0.12	0.48	±	0.12	0.57	±	0.13
Low O2, High NO3	Mixed	A	0.31	±	0.09	0.22	±	0.10	0.25	±	0.06	0.42	±	0.07	0.15	±	0.09	-0.02	±	0.10	0.29	±	0.10	0.38	±	0.11
Low O2, High NO3	Mixed	B	0.30	±	0.08	0.21	±	0.10	0.21	±	0.06	0.38	±	0.08	-0.01	±	0.10	-0.19	±	0.11	0.49	±	0.10	0.59	±	0.12
		**Average**	**0.38**		**0.11**	**0.28**		**0.12**	**0.14**		**0.07**	**0.32**		**0.08**	**0.05**		**0.09**	**-0.13**		**0.10**	**0.43**		**0.12**	**0.53**		**0.13**

Consideration of a lower offset value for nDNF ($\Delta\delta^{15}N$ = 14‰)

			Bacterial Denitrification						Nitrifier Denitrification						Bacterial Ammonia Oxidation						Fungal Denitrification					
Conditions	Site	Replicate	at 10% N_2O reduction			at 90% N_2O reduction			at 10% N_2O reduction			at 90% N_2O reduction			at 10% N_2O reduction			at 90% N_2O reduction			at 10% N_2O reduction			at 90% N_2O reduction		
High NO3	Mud	A	-0.04	±	0.24	-0.30	±	0.30	0.30	±	0.19	0.61	±	0.25	-0.19	±	0.18	-0.50	±	0.24	0.94	±	0.22	1.19	±	0.28
High NO3	Mud	B	0.11	±	0.23	-0.13	±	0.29	0.44	±	0.19	0.74	±	0.25	-0.34	±	0.18	-0.64	±	0.24	0.78	±	0.19	1.02	±	0.26
High NO3	Sand	A	0.22	±	0.15	-0.03	±	0.18	-0.09	±	0.13	0.22	±	0.15	0.29	±	0.13	-0.02	±	0.15	0.57	±	0.15	0.83	±	0.17
High NO3	Sand	B	0.36	±	0.10	0.11	±	0.14	0.04	±	0.08	0.34	±	0.12	0.16	±	0.08	-0.14	±	0.13	0.44	±	0.10	0.69	±	0.14
High NO3	Mixed	A	0.54	±	0.12	0.30	±	0.18	0.31	±	0.10	0.61	±	0.16	-0.11	±	0.08	-0.42	±	0.15	0.26	±	0.10	0.50	±	0.17
High NO3	Mixed	B	0.25	±	0.14	0.00	±	0.20	0.43	±	0.11	0.74	±	0.17	0.06	±	0.11	-0.24	±	0.17	0.26	±	0.14	0.50	±	0.20
Low O2, High NO3	Mud	A	0.43	±	0.13	0.19	±	0.19	0.12	±	0.12	0.43	±	0.17	-0.12	±	0.11	-0.43	±	0.16	0.57	±	0.13	0.81	±	0.19
Low O2, High NO3	Mud	B	0.44	±	0.18	0.19	±	0.23	0.18	±	0.16	0.48	±	0.20	-0.17	±	0.15	-0.48	±	0.19	0.56	±	0.17	0.81	±	0.22
Low O2, High NO3	Mixed	A	0.09	±	0.16	-0.15	±	0.22	0.44	±	0.14	0.75	±	0.19	-0.05	±	0.13	-0.35	±	0.19	0.50	±	0.15	0.76	±	0.21
Low O2, High NO3	Mixed	B	0.12	±	0.17	-0.13	±	0.23	0.38	±	0.15	0.69	±	0.20	-0.18	±	0.14	-0.48	±	0.20	0.68	±	0.15	0.93	±	0.22
		**Average**	**0.25**		**0.16**	**0.00**		**0.22**	**0.26**		**0.14**	**0.56**		**0.19**	**-0.06**		**0.13**	**-0.37**		**0.18**	**0.56**		**0.15**	**0.80**		**0.21**

Supplementary Table 6. Sensitivity analysis of fractional estimates of N_2O production calculated by isotope mass balance. Error estimated by Monte Carlo simulation. See text for details. Compare to table S4 (base case) for relative magnitude of changes.

Consideration of fDNF having a lower SP of 30.3‰ +/- 4.8

			Bacterial Denitrification		Nitrifier Denitrification		Bacterial Ammonia Oxidation		Fungal Denitrification	
**Conditions**	**Site**	**Replicate**	at 10% N_2O reduction	at 90% N_2O reduction	at 10% N_2O reduction	at 90% N_2O reduction	at 10% N_2O reduction	at 90% N_2O reduction	at 10% N_2O reduction	at 90% N_2O reduction
High NO3	Mud	A	0.08 ± 0.24	0.07 ± 0.23	0.08 ± 0.05	0.17 ± 0.05	0.03 ± 0.12	-0.06 ± 0.12	0.82 ± 0.27	0.83 ± 0.26
High NO3	Mud	B	0.39 ± 0.11	0.38 ± 0.11	0.12 ± 0.04	0.21 ± 0.05	-0.02 ± 0.17	-0.11 ± 0.18	0.51 ± 0.22	0.51 ± 0.23
High NO3	Sand	A	0.04 ± 0.15	0.03 ± 0.15	-0.04 ± 0.04	0.05 ± 0.04	0.24 ± 0.10	0.15 ± 0.10	0.76 ± 0.18	0.77 ± 0.18
High NO3	Sand	B	0.33 ± 0.09	0.32 ± 0.09	0.00 ± 0.03	0.09 ± 0.03	0.20 ± 0.05	0.11 ± 0.04	0.47 ± 0.10	0.48 ± 0.10
High NO3	Mixed	A	0.79 ± 0.09	0.78 ± 0.09	0.09 ± 0.02	0.18 ± 0.02	0.11 ± 0.06	0.02 ± 0.06	0.01 ± 0.11	0.02 ± 0.11
High NO3	Mixed	B	0.61 ± 0.12	0.61 ± 0.12	0.13 ± 0.03	0.22 ± 0.03	0.37 ± 0.03	0.28 ± 0.03	-0.11 ± 0.12	-0.11 ± 0.12
Low O2, High NO3	Mud	A	0.45 ± 0.09	0.45 ± 0.09	0.03 ± 0.03	0.12 ± 0.04	-0.02 ± 0.07	-0.12 ± 0.07	0.54 ± 0.12	0.55 ± 0.11
Low O2, High NO3	Mud	B	0.51 ± 0.09	0.50 ± 0.09	0.04 ± 0.04	0.13 ± 0.05	-0.04 ± 0.11	-0.13 ± 0.11	0.49 ± 0.15	0.50 ± 0.15
Low O2, High NO3	Mixed	A	0.43 ± 0.08	0.42 ± 0.08	0.13 ± 0.03	0.22 ± 0.03	0.27 ± 0.09	0.18 ± 0.08	0.17 ± 0.13	0.18 ± 0.12
Low O2, High NO3	Mixed	B	0.36 ± 0.07	0.35 ± 0.07	0.10 ± 0.03	0.19 ± 0.03	0.10 ± 0.10	0.01 ± 0.10	0.44 ± 0.13	0.45 ± 0.13
		**Average**	**0.40 0.11**	**0.39 0.11**	**0.07 0.04**	**0.16 0.04**	**0.12 0.09**	**0.03 0.09**	**0.41 0.15**	**0.42 0.15**

Consideration of nDNF having access to extracellular nitrite ($\Delta^{17}_{N2O}/\Delta^{17}_{NO2} = 1$)

			Bacterial Denitrification		Nitrifier Denitrification		Bacterial Ammonia Oxidation		Fungal Denitrification	
**Conditions**	**Site**	**Replicate**	at 10% N_2O reduction	at 90% N_2O reduction	at 10% N_2O reduction	at 90% N_2O reduction	at 10% N_2O reduction	at 90% N_2O reduction	at 10% N_2O reduction	at 90% N_2O reduction
High NO3	Mud	A	0.17 ± 0.18	0.14 ± 0.18	0.11 ± 0.05	0.21 ± 0.05	0.10 ± 0.13	0.10 ± 0.13	0.62 ± 0.24	0.55 ± 0.24
High NO3	Mud	B	0.43 ± 0.10	0.41 ± 0.10	0.15 ± 0.04	0.26 ± 0.05	0.10 ± 0.22	0.10 ± 0.21	0.31 ± 0.22	0.23 ± 0.22
High NO3	Sand	A	0.15 ± 0.12	0.13 ± 0.11	-0.03 ± 0.04	0.08 ± 0.04	0.20 ± 0.11	0.20 ± 0.11	0.68 ± 0.16	0.60 ± 0.16
High NO3	Sand	B	0.39 ± 0.07	0.37 ± 0.07	0.01 ± 0.03	0.12 ± 0.03	0.20 ± 0.04	0.20 ± 0.04	0.40 ± 0.08	0.32 ± 0.08
High NO3	Mixed	A	0.76 ± 0.08	0.72 ± 0.09	0.11 ± 0.03	0.23 ± 0.04	0.20 ± 0.08	0.20 ± 0.08	-0.07 ± 0.11	-0.15 ± 0.11
High NO3	Mixed	B	0.56 ± 0.11	0.53 ± 0.11	0.15 ± 0.04	0.26 ± 0.05	0.50 ± 0.02	0.50 ± 0.02	-0.22 ± 0.12	-0.30 ± 0.12
Low O2, High NO3	Mud	A	0.52 ± 0.07	0.49 ± 0.07	0.05 ± 0.03	0.15 ± 0.03	0.00 ± 0.07	0.00 ± 0.07	0.43 ± 0.09	0.36 ± 0.10
Low O2, High NO3	Mud	B	0.56 ± 0.08	0.54 ± 0.08	0.06 ± 0.05	0.17 ± 0.05	0.00 ± 0.13	0.00 ± 0.13	0.37 ± 0.13	0.29 ± 0.13
Low O2, High NO3	Mixed	A	0.42 ± 0.08	0.39 ± 0.08	0.15 ± 0.04	0.26 ± 0.04	0.40 ± 0.10	0.40 ± 0.10	0.03 ± 0.12	-0.05 ± 0.12
Low O2, High NO3	Mixed	B	0.40 ± 0.05	0.37 ± 0.06	0.13 ± 0.03	0.23 ± 0.04	0.20 ± 0.12	0.20 ± 0.11	0.27 ± 0.12	0.20 ± 0.12
		**Average**	**0.44 0.09**	**0.41 0.09**	**0.09 0.04**	**0.20 0.04**	**0.19 0.10**	**0.19 0.10**	**0.28 0.14**	**0.20 0.14**
